# Host Glycan Sugar-Specific Pathways in *Streptococcus pneumonia*: Galactose as a Key Sugar in Colonisation and Infection

**DOI:** 10.1371/journal.pone.0121042

**Published:** 2015-03-31

**Authors:** Laura Paixão, Joana Oliveira, André Veríssimo, Susana Vinga, Eva C. Lourenço, M. Rita Ventura, Morten Kjos, Jan-Willem Veening, Vitor E. Fernandes, Peter W. Andrew, Hasan Yesilkaya, Ana Rute Neves

**Affiliations:** 1 Instituto de Tecnologia Química e Biológica, Universidade Nova de Lisboa, Oeiras, Portugal; 2 Centre for Intelligent Systems, LAETA, IDMEC, Instituto Superior Técnico, Universidade de Lisboa, Lisboa, Portugal; 3 Molecular Genetics Group, Groningen Biomolecular Sciences and Biotechnology Institute, Centre for Synthetic Biology, University of Groningen, Groningen, The Netherlands; 4 Department of Infection, Immunity & Inflammation, University of Leicester, Leicester, United Kingdom; Rockefeller University, UNITED STATES

## Abstract

The human pathogen *Streptococcus pneumoniae* is a strictly fermentative organism that relies on glycolytic metabolism to obtain energy. In the human nasopharynx *S*. *pneumoniae* encounters glycoconjugates composed of a variety of monosaccharides, which can potentially be used as nutrients once depolymerized by glycosidases. Therefore, it is reasonable to hypothesise that the pneumococcus would rely on these glycan-derived sugars to grow. Here, we identified the sugar-specific catabolic pathways used by *S*. *pneumoniae* during growth on mucin. Transcriptome analysis of cells grown on mucin showed specific upregulation of genes likely to be involved in deglycosylation, transport and catabolism of galactose, mannose and N acetylglucosamine. In contrast to growth on mannose and N-acetylglucosamine, *S*. *pneumoniae* grown on galactose re-route their metabolic pathway from homolactic fermentation to a truly mixed acid fermentation regime. By measuring intracellular metabolites, enzymatic activities and mutant analysis, we provide an accurate map of the biochemical pathways for galactose, mannose and N-acetylglucosamine catabolism in *S*. *pneumoniae*. Intranasal mouse infection models of pneumococcal colonisation and disease showed that only mutants in galactose catabolic genes were attenuated. Our data pinpoint galactose as a key nutrient for growth in the respiratory tract and highlights the importance of central carbon metabolism for pneumococcal pathogenesis.

## Introduction

*Streptococcus pneumoniae* is an important human pathogen responsible for high morbidity and mortality worldwide, mainly due to community-acquired pneumonia, meningitis, bacteraemia and otitis media [1,2]. The pneumococcus is, however, also a transient commensal that asymptomatically resides and proliferates in the human nasopharynx [3]. Colonisation of the nasopharynx is of importance as it represents a reservoir from which bacteria can disseminate throughout the community [2,4]. Furthermore, the establishment of a carrier state is accepted to be a pre-requisite for disease [3,5]. Despite the significance of colonisation to the lifestyle of *S*. *pneumoniae*, little is known about the mechanisms employed by the bacterium to grow and proliferate in the human nasopharynx.

*S*. *pneumoniae* is a strictly fermentative bacterium that relies on glycolytic metabolism to obtain energy [6]. Therefore, the ability to acquire and metabolize sugars is of major importance for *in vivo* fitness of this microorganism. Indeed, the pneumococcus lacks a complete set of genes for respiratory proteins and is for this reason unable to generate energy by respiration [6,7]. The genomic abundance of genes involved in sugar transport further supports the significant role of carbohydrates in the lifestyle of *S*. *pneumoniae* [6,7]. Over 30% of the transporters in the *S*. *pneumoniae* genome were predicted to be involved in the uptake of carbohydrates [7], and these predictions were validated by a recent functional genomic approach targeting carbohydrate transport [8]. Furthermore, several putative carbohydrate degradation pathways have been annotated in the genome sequences of *S*. *pneumoniae* [6,7]. In summary, *S*. *pneumoniae* potentially harbours an incredible flexibility with respect to sugar consumption. Hexoses, and in particular glucose (Glc), are the preferred carbon and energy sources of *S*. *pneumoniae* [9]. However, in the human airway the abundance of free sugars is scarce, the concentration of Glc being below 1 mM [10,11]. In this case, *in vivo* growth must rely on alternative nutritional reservoirs. In the human nasopharynx, the glycoproteins lining the epithelial surfaces appear as good candidates to serve as carbon and energy sources for pneumococcal growth. Importantly, *S*. *pneumoniae* is able to grow on mucin as a sole carbon source [12]. Mucins are major components of the mucus that cover the epithelial surfaces [13]. These structures are heavily O-glycosylated glycoproteins and despite their composition variability, mucins are generally composed of N-acetylglucosamine (GlcNAc), N-acetylgalactosamine (GalNAc), N-acetylneuraminic acid (NeuNAc), galactose (Gal), fucose (Fuc) and sulphated sugars linked to the protein core, most commonly via a N-acetylgalactosamine moiety [13,14]. Furthermore, *S*. *pneumoniae* can use other host glycans, such as N-glycans and glycosaminoglycans [15,16]. In fact, the pneumococcus is equipped with a high number of extracellular glycosidases covering a wide range of substrates specificities (reviewed in [17]). The action of these enzymes on host glycans generates a variety of free sugars that potentially can be used by the pneumococcus. The deglycosylation activity of both exo- and endoglycosidades has been previously demonstrated in *S*. *pneumoniae* [15,18–20]. Furthermore, the role of these enzymes in *in vivo* fitness is substantiated by the observations that glycosidase mutants show attenuated ability to colonise and to cause disease in mouse models [14,19–23].

In this study, we set out to identify the putative catabolic pathways important for growth on mucin. To this end, we used the well-established laboratorial model for pneumococcal studies *S*. *pneumoniae* D39 [24]. Subsequently, the predicted utilization routes of the glycan-derived sugars Gal, GlcNAc and Man were functionally established, by positive detection of phosphorylated metabolic intermediates and measurement of specific enzyme activities. Inactivation of a unique gene in each catabolic pathway rendered mutant strains unable or with impaired ability to grow in the presence of the corresponding sugar. Finally, the contribution of the sugar-specific catabolic pathways to colonisation and pneumococcal disease was assessed in appropriate mouse models, demonstrating that mutants in Gal catabolic pathways were attenuated in their ability to colonise and had reduced virulence in respiratory infection models.

Overall, we experimentally validated the catabolic pathways of glycan-derived Gal, Man and GlcNAc and found Gal as a key nutrient during pneumococcal *in vivo* growth.

## Materials and Methods

### Bacterial strains and growth conditions

*Streptococcus pneumoniae* strain D39 (serotype 2) and its derivatives are listed in S1 Table. The D39 isolate was obtained from the culture collection of the Department of Infection, Immunity and Inflammation, of the University of Leicester. Stocks and working stocks were prepared as described elsewhere [25] and stored in 25% (vol/vol) glycerol M17 medium (Difco) at -80°C.

Routinely, *S*. *pneumoniae* was grown statically in M17 broth containing 0.5% (wt/vol) glucose (Glc-M17) at 37°C. For physiological studies, bacteria were grown in static rubber-stoppered bottles (80 ml in 100 ml bottles) at 37ºC and without pH control (initial pH 6.5) in the chemically defined medium (CDM) described by Carvalho *et al*. [25]. For each sugar tested (Gal, GlcNAc, Man and Glc), growth was analysed under sugar excess (34±2 mM) and a lower concentration (13±1 mM). In sugar mixtures each carbohydrate (Gal, Man and GlcNAc) was added to an initial concentration of *circa* 6.5 mM. Cultures were started by inoculating fresh CDM, to an initial optical density at 600 nm (OD_600_) of ~0.05, with a pre-culture grown until late-exponential phase of growth. Pre-cultures were performed as described previously [25]. Pre-cultures for growth on sugar mixtures were grown in CDM containing 30 mM of each carbon source. Growth was monitored by measuring OD_600_ hourly. Maximum specific growth rates (μ_max_) were calculated through linear regressions of the plots of ln(OD_600_) *versus* time during the exponential phase of growth. The values reported are averages of at least eight independent growths.

Representative growth curves were selected based on the minimum value of the sum:

$${\left(\frac{OD_{\max}-{\bar{OD}}_{\max}}{SD_{OD\max}}\right)}^{2}+{\left(\frac{\mu_{\max}-{\bar{\mu }}_{\max}}{SD_{\mu \max}}\right)}^{2}$$

Where, ${\bar{OD}}_{\max}$ and ${\bar{\mu }}_{\max}$ are the averages of the maximum optical density (OD_max_) and μ_max_, respectively; *SD*_*OD*max_ and *SD*_*μ*max_ are the standard deviation of OD_max_ and μ_max_, respectively.

### Statistical analysis of the growth parameters

ANOVA was applied to test the hypothesis that *μ*_max_ values are independent of the sugar. Additionally, the differences across the two initial substrate conditions were also compared. The same ANOVA procedure was taken to assess if differences of OD_max_ values depended on the sugar and initial condition and assess if they were statistically significant.

The null hypothesis of equal values for the μ_max_ was tested for all possible pairwise combinations of initial conditions and sugars. This was accomplished by independent two-sample t-tests, whose results are summarized on S2 Table. Likewise, similar tests were also performed for the OD_max_. Results are presented in S2 Table.

### Multiple non-linear regression method for generating confidence interval bands

Multiple non-linear regressions were performed for all the combinations of experimental conditions (4 sugars and 2 initial concentrations) using the Gompertz model [26]:

$$f(t;\mu_{\max},\lambda ,A)=A\cdot \exp\left(-\exp\left\{\frac{\mu_{\max}\cdot \exp(1)}{A}\left(\lambda -t\right)+1\right\}\right),$$

where μ_max_ is the tangent in the inflection point (maximum growth rate), *λ* is the x-axis intercept of this tangent (lag) and *A* is the asymptote $A=\log\left(\frac{y_{\infty }}{y_{0}}\right)$. To obtain better fittings, the data were previously log-transformed as $y^{\prime }=\log\left(\frac{y}{y_{0}}\right)$ and the parameters estimated directly with the BGFit web-application [27] using non-linear least squares.

After obtaining the estimates for each sugar and initial concentration condition, a 95% confidence interval for the data and bands for the predicted responses of the model were computed. These computations were performed in MATLAB and Statistics Toolbox R2013a using the function *nlpredci*.

### General molecular techniques

Chromosomal DNA isolation was performed according to the procedure described by Johansen and Kibenich [28]. Pwo polymerase was used according to the supplier’s instructions (Roche). PCRs were performed with a MyCycler thermal cycler (Bio-Rad). Purification of the PCR fragments was accomplished using the High Pure PCR product Purification Kit (Roche) according to the supplier’s instructions. Plasmid isolation was done using a High Pure Plasmid Isolation Kit (Roche), according to the manufacturer’s protocol. Restriction enzymes were purchased from New England Biolabs.

### Construction of loss-of-function mutants

Chromosomal DNA of *S*. *pneumoniae* D39 was used as template in the PCR amplifications. Oligonucleotide primers used for these constructs are listed in S3 Table. *galK* (SPD_1634), *lacD* (SPD_1050), *manA* (SPD_0641), *nagA* (SPD_1866) and *galT-2* (SPD_1633) disruption was accomplished by allelic replacement mutagenesis, essentially as described by Song *et al*. [29]. The upstream and downstream flanking regions of the genes to be disrupted were amplified using the appropriate primers’ combinations KO1_Fw/KO2_Rv_Spe and KO3_Fw_Spe/KO4_Rv, respectively (S3 Table). Flanking fragments were fused to the spectinomycin resistance marker (Spe) (1032 bp, amplified with primers Spe_Fp and Spe_Rp from pORI38), by overlap extension PCR using the appropriate primers KO1_Fw and KO4_Rv. The resulting fused fragments were purified and transformed into D39 as described before [30]. Positive transformants were selected on Glc-M17 sheep blood (1% vol/vol) agar plates supplemented with 150 μg ml^-1^ of spectinomycin. The correct integration of the insert in the mutant clones was confirmed by PCR. Genomic DNA was used as template for PCR with primers designed to anneal around 100 bp upstream and downstream of the recombination site, as well as combinations of these primers with those used to construct the mutants (S3 Table).

A double mutant, D39Δ*lacD*Δ*galK*, was constructed by allelic replacement of the *galK* gene in the D39Δ*lacD* mutant using trimethoprim (Tmp) selection. The up and downstream flanking regions of the *galK* gene were amplified using the appropriate primer combinations: GalK_KO1_Fw/GalK_KO2_Rv_Tmp and GalK_KO3_Fw_Tmp/GalK_KO4_Rv, respectively (S3 Table). The regions flanking *galK* were fused to the Tmp cassette in an overlap extension PCR reaction with the primer combination GalK_KO1_Fw/GalK_KO4_Rv, yielding Δ*galK*::*tmp*. The purified fused fragment was transformed into D39Δ*lacD* and positive clones were selected on Glc-M17 sheep blood (1% vol/vol) agar plates supplemented with 18 μg ml^-1^ of trimethoprim. Gene replacement was confirmed as described above, using the primers listed in S3 Table.

### Construction of the pKB01 derivatives for complementation studies

pKB01, containing a zinc-inducible P_*czcD*_ promoter (P_Zn_), was used as a complementation system [31]. The target genes were amplified using chromosomal DNA from *S*. *pneumoniae* D39. For the construction of pKB01-*lacD*, pKB01-*galK* and pKB01-*manA*, *lacD*, *galK* and *manA* genes were amplified using LacD_Fw_EcoRI/LacD_Rv_BamHI, GalK_Fw_EcoRI/GalK_Rv_BamHI and ManA_Fw_EcoRI/ManA_Rv_BamHI, respectively. The PCR-amplified fragments and pKB01 were digested with EcoRI and BamHI and subsequently ligated. To generate pKB01-*nagA* the gene was amplified with its own promoter using primers NagA_Fw_NotI/NagA_Rv_BamHI. The digested fragment (NotI/BamHI) was cloned into pKB01 using the same restriction sites. pKB01-*galT-2* was made by amplifying *galT-2* with GalT-2_Fw_EcoRI/GalT-2_Rv_NotI. The PCR-fragment and PKB01 were cleaved using EcoRI/NotI enzymes and ligated. To construct PKB01-*galKgalT-2*, *galKgalT-2* was amplified using GalK_Fw_EcoRI_B/GalT-2_Rv_XbaI, digested with EcoRI and XbaI and ligated into pKB01 at the same restriction sites. The primers used are listed in S3 Table.

All the generated constructs were transformed into *E*. *coli* DH5α [32]. *E*. *coli* was grown in Luria broth at 37ºC supplemented with 100 μg ml^-1^ ampicillin. The constructs were verified by sequencing at Macrogen.

### Complementation of deletion strains

The pKB01-based plasmids were transformed into competent cells of *S*. *pneumoniae* D39 loss-of-function mutants. For transformation, 2 μl of the competence-stimulating peptide (CSP, 0.1 mg ml^-1^) was added to pre-competent cells and activation achieved by 12 min at 37ºC. Plasmid DNA was added and transformation was accomplished by 20 min at 30ºC, followed by a phenotypic expression period of 90 min at 37ºC and overnight growth on Columbia blood agar plates supplemented with 1 μg ml^-1^ of tetracycline [33]. pKB01 constructs integrate by a double cross-over into the chromosomal *bgaA* locus. Correct integration was verified, in single colonies, by PCR. The constructed strains are listed in S1 Table.

### Growth of complemented strains

Growth experiments were performed in a 96 well microtiter plate reader (Tecan Genius) in a total volume of 200 μl C+Y medium [34], devoid of Glc and sucrose. The medium was supplemented with 55 mM of the desired carbon source in the presence or absence of 0.1 mM ZnCl_2_. Cells were grown at 37ºC and OD_595_ was measured hourly.

### Transcriptome analysis

For microarrays analysis, *S*. *pneumoniae* D39 was grown using Sicard’s defined medium with or without modification [35]. Modification was done to include mucin as the sole carbon source, replacing Glc and bovine serum albumin. Porcine gastric mucin (Sigma) was dissolved in water at a concentration of 10 mg ml^-1^ and dialysed against water overnight at 4ºC using snake skin dialysis membrane (MWCO 10 kDa, Pierce). After freeze drying, the mucin was dissolved in 10 mM potassium phosphate buffer, pH 7.0, autoclaved at 121ºC for 15 min. This was then briefly centrifuged to remove insoluble residues and mixed with 2X concentrated Sicard’s medium. The extraction of RNA was done as described previously [36,37].

### Microarray experiments

Microarray slides were purchased from the Bacterial Microarray Group at St. George’s Hospital Medical School, University of London. The SPv1.1.0 array contained spotted PCR products that represent all of the genes in the *S*. *pneumoniae* TIGR4 and R6 genomes. The array design is available in BμG@Sbase (accession number: A-BUGS-14; http://bugs.sgul.ac.uk/A-BUGS-14) and also ArrayExpress (accession number: A-BUGS-14). The Materials and Methods for microarray analysis followed previously reported methodology [37].

### Analysis of microarrays

The microarray slides were scanned using an Axon GenePix 4000A microarray scanner, which utilises GenePix 5.1 software (Molecular Devices Ltd) for identification and for a visual analysis of the quality of the spots. The raw intensity data obtained from four independent experiments were normalised and further analysed using GeneSpring 7.3 software (Agilent Technologies). Data were subjected to LOWESS intensity-dependent normalisation. Statistically significant changes in gene expression were determined as t-test *p*-values < 0.05 after Benjamini and Hochberg false discovery rate correction [38]. Genes of interest were further identified by requiring >2-fold differences in all four samples analysed. In addition, the microarray results for selected genes whose expression significantly altered in the presence of mucin were verified and confirmed by real time quantitative reverse transcription PCR (qRT-PCR), in order to ensure that dye affinity did not bias the results.

Fully annotated microarray data have been deposited in BμG@Sbase (accession number E-BUGS-159; http://bugs.sgul.ac.uk/E-BUGS-159) and also ArrayExpress (accession number E-BUGS-159).

### Quantitative RT-PCR

To assess the expression of specific Gal catabolic genes (*galT-2*, *galT-1*, *galK*, *lacD*), by qRT-PCR, cells were grown in CDM supplemented with the appropriate carbohydrate, as previously described, and a 4 ml aliquot was collected (16,100 *x g*, 2 min at 27ºC) in mid-exponential phase of growth. The supernatant was discarded and the pellet suspended in 0.5 ml Trizol (Life Technologies). Samples were stored at -80ºC until further analysis. RNA extraction was performed as described previously [37].

To confirm the microarray results, two independent RNA preparations were used for qRT-PCR analysis. First strand cDNA synthesis was performed on approximately 1 μg DNase-treated total RNA, immediately after isolation, using 200 U of SuperScript II reverse transcriptase (Invitrogen) and random hexamers at 42°C for 55 min [39]. cDNA (15 ng) was amplified in a 20 μl reaction volume that contained 1 x SYBR Green PCR master mix (Applied Biosystems) and 3 pmol of each primer (S3 Table). The transcription level of genes was normalised to *gyrB* transcription, amplified in parallel with SPDRT0709F and SPDRT0709R primers. The reactions were performed in triplicate using the following cycling parameters with a Rotor Gene real time PCR cycler (Qiagen): 1 cycle of 10 min 95ºC followed by 40 cycles of 30 sec 95ºC, 1 min 55°C, and 30 sec 72°C. The results were interpreted using the comparative C_T_ method [40].

### *In silico* analysis for catabolic pathway prediction

Potential catabolic pathways for the monosaccharides Gal, Man, GlcNAc, NeuNAc, GalNAc and Fuc were predicted *in silico* by combining data obtained from the metabolic databases MetaCyc (http://metacyc.org/META/class-tree?object=Pathways) and Kegg (http://www.genome.jp/kegg/pathway.html), literature surveys and genome sequences. In addition, functionally characterized proteins from other microorganisms were used as query for BlastP searches of *S*. *pneumoniae* D39 genome deposited at NCBI (http://www.ncbi.nlm.nih.gov/genome/176?project_id=58581).

### Growth assay for assessment of sugar utilization by *S*. *pneumoniae* D39

The ability of monosaccharides Glc, Gal, Man, GlcNAc, glucosamine (GlcN), galactosamine (GalN), and GalNAc to support growth of *S*. *pneumoniae* D39 was investigated using 96-well microtiter plates containing 250 μl CDM supplemented with 30 mM of each sugar. Cultures were started at an initial OD_595_ of ~0.05 by addition of an overnight Glc-grown pre-culture pelleted (6300 *x g*, 7 min, RT) and suspended in fresh CDM without sugar. Growth was monitored at 595 nm over 24 h at 37ºC. Readings were taken every 30 min, after 1 s shaking, using an ELx808 Absorbance Microplate Reader (BioTek Instruments, Inc.). The growth curves were generated by using Gen5^TM^ (BioTek Instruments, Inc.). Each growth condition was done in triplicate using two independent pre-cultures.

### Quantification of sugar consumption and fermentation products

Strains were grown in CDM supplemented with the appropriate sugar as described above. Culture samples (2 mL) were taken immediately after inoculation and at the onset of the stationary phase of growth, and centrifuged (16,100 *x g*, 3 min, 4ºC). For high performance liquid chromatography (HPLC) analysis, samples were treated as described by Carvalho *et al*. [9]. Fermentation products and Glc were quantified by HPLC as before [9]. Gal, Man, GlcNAc and formate were quantified by ^1^H-NMR and the spectra were acquired with a Bruker AMX300 spectrometer (Bruker BioSpin GmbH). To quantify Gal the temperature of the probe was set to 18ºC, whereas for Man and GlcNAc it was 37ºC. DSS (3-(trimethylsilyl) propionic acid sodium salt) was added to the samples as an internal concentration standard in ^1^H-NMR quantifications.

Yields were calculated using the data from samples taken immediately after inoculation and at the onset of stationary phase of growth. A factor of 0.38, determined from a dry weight (DW) (mg ml^-1^) *versus* OD_600_ curve, was used to convert OD_600_ into DW (mg biomass ml^-1^). The yield in biomass was calculated as g of dry weight per mol of substrate consumed. The ATP yield was determined as the ratio of ATP produced to substrate consumed at the time of growth arrest assuming that all ATP was synthesized by substrate-level phosphorylation. The values reported are averages of at least two independent growths.

### Cold ethanol extractions and determination of intracellular metabolites by ^31^P-NMR

For ethanol extractions, the Na_2_-β-glycerophosphate in the CDM growth medium was replaced by 15.4 g l^-1^ MES (2-(N-morpholino) ethanesulfonic acid), to avoid the intense buffer resonance in the phosphomonoester (PME) region. The ethanol extracts were prepared as described previously by Carvalho *et al*. [9]. Cells were harvested (20,980 *x g*, 4ºC, 5 min) during exponential growth and the pellet suspended in milliQ water, pH 6.5. Cell suspensions were transferred to the appropriate volume of cold ethanol 70% (vol/vol) in an ice bath, and extraction was performed for 30 min with vigorous agitation. Cell debris was removed by centrifugation (39,191 *x g*, 4ºC, 20 min). The ethanol in the supernatant was removed via a rotavap and the extract frozen in liquid nitrogen, and lyophilized overnight. The dried extract was dissolved in 1 ml of deuterated water containing 5 mM EDTA. The pH was set to 6.5 and the extract was stored at -20ºC until analysis by ^31^P-NMR. Resonances were assigned by addition of pure compounds to the extracts or on basis of comparison with previous studies [9]. ^31^P-NMR spectra were recorded using a selective probe head (^31^P-SEX) at 30ºC on a Bruker AVANCE II 500 MHz spectrometer (Bruker BioSpin GmbH) by using standard Bruker pulse programs. Spectra were referenced to the resonance of external 85% H_3_PO_4_, designated at 0 ppm.

### Enzyme activity determination

For enzyme activity determination, cells of *S*. *pneumoniae* were grown, in CDM supplemented with 30 mM sugar (Gal, Man, GlcNAc or Glc), until late-exponential phase of growth, and harvested by centrifugation (7,519 *x g*, 7 min, 4ºC). The supernatant was removed, the pellet suspended in cold potassium phosphate buffer (KP_i_) 10 mM, pH 7.0, and stored at -20ºC until further analysis. Dithiothreitol (1 mM) was added to the suspension and cell-free extracts prepared by mechanical disruption in a French Press (6.21 MPa). Cell debris was removed (16,100 *x g* for 15 min, at 4ºC) and the supernatant was used for enzyme activity measurements. Extracts were kept on ice during these measurements.

For measurement of galactokinase (GalK) activity, removal of low molecular weight substances from the cell-free extract was performed using a PD-10 desalting column equilibrated with KP_i_ buffer 10 mM pH 7.0 according to the supplier’s instructions (GE Healthcare Life Sciences). The GalK assay mixture contained 100 mM triethanolamine (TEA) buffer pH 7.6, 5 mM MgCl_2_, 10 mM ATP and 10 mM galactose. The mixture was incubated at 37ºC, in a Thermomixer comfort (Eppendorf) and the reaction started by the addition of the cell-free extract. The reaction was stopped, at different time points, by incubating for 5 min at 85ºC and subsequently freezing in liquid nitrogen. Samples were stored at -20ºC until further analysis. Quantification of galactose 1-phosphate formed was accomplished by ^1^H-NMR spectroscopy using a Brucker AMX300 spectrometer (Brucker BioSpin GmbH). DSS was added to the samples and used as an internal concentration standard. The slopes of the galactose 1-phosphate (Gal1P) formed *versus* time were determined using linear regression.

Tagatose 1,6-diphosphate aldolase (LacD) activity was determined essentially as described by Crow and Thomas [41]. The reaction mixture (1 ml) contained 50 mM TEA buffer pH 7.8, 0.25 mM NADH, 1.2 U α-glycerolphosphate dehydrogenase (Roche), 11.5 U triosephosphate isomerase from rabbit muscle (Sigma-Aldrich) and 0.16 mM tagatose 1,6-diphosphate (TBP). The oxidation of NADH was measured by the decrease in absorbance at 340 nm.

N-acetylglucosamine 6-phosphate deacetylase (NagA) activity was enzymatically assayed as described by Homer *et al*. [42]. The method couples this activity to the other GlcNAc-specific catabolic enzyme—glucosamine 6-phosphate isomerase (NagB). To ensure the applicability of this protocol, the specific activity of NagB was measured [43]. The specific activity of this enzyme was higher than that of NagA for all conditions assayed, thus showing that the glucosamine 6-phosphate isomerase activity was not a rate limiting step. The reaction mixture (1 ml) contained 40 mM sodium phosphate buffer pH 7.5, 1 mM N-acetylglucosamine 6-phosphate, 0.2 mM NADP^+^, 4 U phosphoglucose isomerase (Sigma) and 1.5 U glucose 6-phosphate dehydrogenase (Roche). Glucosamine 6-phosphate isomerase was measured in an identical assay but N-acetylglucosamine 6-phosphate was replaced by glucosamine 6-phosphate. NADPH formation was monitored by measuring the increase in absorbance at 340 nm spectrophotometrically.

Mannose 6-phosphate isomerase (ManA) activity was measured essentially as described by Gracy and Noltmann [44], and modified as follows: the assay mixture contained 100 mM TEA pH 7.6, 10 mM mannose 6-phosphate, 2 mM NADP^+^, 1 U of phosphoglucose isomerase (Sigma) and 1 U of glucose 6-phosphate dehydrogenase (Roche), in a total volume of 250 μl. The rate of change of absorbance at 340 nm (due to NADP^+^ reduction), coupled to mannose 6-phosphate isomerization, was measured spectrophotometrically.

The coupled enzyme protocols were carried out at 25ºC. All the reactions were started by adding adequate amounts of freshly prepared cell-free extracts. The absorbance changes were recorded in a Shimadzu UV-1603 spectrophotometer (Shimadzu Corporation).

One unit (U) of GalK activity is defined as the amount of protein required for the formation of 1 μmol of Gal1P per minute. For the coupled enzyme protocols, the enzyme activity is given as the amount of protein required to catalyse the oxidation or reduction of 1 μmol of NADH or NADP^+^, respectively, per minute. Specific activity was expressed as units (μmol min^-1^) per milligram of protein (U mg protein^-1^). Protein concentration in the cell-free extracts was determined by the Pierce BCA protein assay kit (Thermo Scientific). All the determinations were made at least in triplicate in two extracts obtained from independent cultures.

### *In vivo* analysis of pneumococcal strains

Ten-week-old female MF1 outbred mice (Charles River) were used. Before use, a standard inoculum for each pneumococcal strain was prepared as described before [39].

To assess the virulence of pneumococcal strains, mice were lightly anesthetized with 3% (vol/vol) isoflurane over oxygen, and an inoculum of 50 μl containing approximately 1 X 10^6^ CFU in PBS was given drop by drop into the nostrils. After infection, the inoculum dose was confirmed by viable counting on blood agar plates. Animals were monitored for disease signs (progressively starry coat, hunched, and lethargic) every six hours in the first 24 h. After the onset of disease signs, which is after 24 h post-infection, the mice were monitored every 2 hours [39,45]. When the mice become lethargic, they were culled by cervical dislocation. Therefore, time to reach lethargic state was defined as the “survival time.” Mice that were alive 7 days after infection were deemed to have survived the infection.

Colonisation experiments were done essentially as described above except that mice were administered with 5 X 10^5^ CFU of *S*. *pneumoniae* in 10 μl PBS. For intravenous infections, approximately 5 X 10^5^ CFU of *S*. *pneumoniae* in 100 μl PBS (pH 7.0) were administered via a tail vein. The inoculum dose was confirmed by plating onto blood agar, as described above.

To monitor the development of bacteraemia, approximately 20 μl of venous blood was obtained from each mouse at predetermined time points after infection, and viable counts were determined, as described above. The growth of pneumococci in the nasopharynx was also determined, as described previously [39,45]. For this, at predetermined time intervals following intranasal infection, pre assigned groups of mice were deeply anesthetized with 5% (vol/vol) isoflurane over oxygen, and the mice were subsequently killed by cervical dislocation. Nasopharyngeal tissue was collected as described previously [39,45] and transferred into 10 ml of sterile PBS, weighed, and then homogenized with an Ultra Turrax blender (Ika-Werke). Viable counts in homogenates were determined as described above.

Survival times were calculated by using GraphPad Prism software and analysed by the Mann-Whitney U test. Data were analysed by an analysis of variance followed by the Bonferroni post-test. Statistical significance was considered to be a *p*-value of < 0.05.

### Chemicals

N-acetylglucosamine 6-phosphate (GlcNAc6P) was obtained through a modification of established procedures [46,47] according to S1 Fig The synthesis of tagatose 1,6-diphosphate (TBP) is described in S2 Fig Modifications to the synthesis of TBP previously published [48] were made in order to optimize the process (see S1 Text). The synthesized compounds were quantified by ^1^H-NMR, using a Brucker AMX300 spectrometer (Brucker BioSpin GmbH). DSS, used as an internal concentration standard in ^1^H-NMR quantifications, was purchased from Merck.

Galactose, mannose, and N-acetylneuraminic acid were purchased from Sigma-Aldrich. Glucose was supplied by Merck and N-acetyl-D-galactosamine, N-acetyl-D-glucosamine, glucosamine and galactosamine were purchased from Applichem. All other chemicals used were reagent grade.

## Results

### Genomic potential for the utilization of host monosaccharides

**Fig 1:**
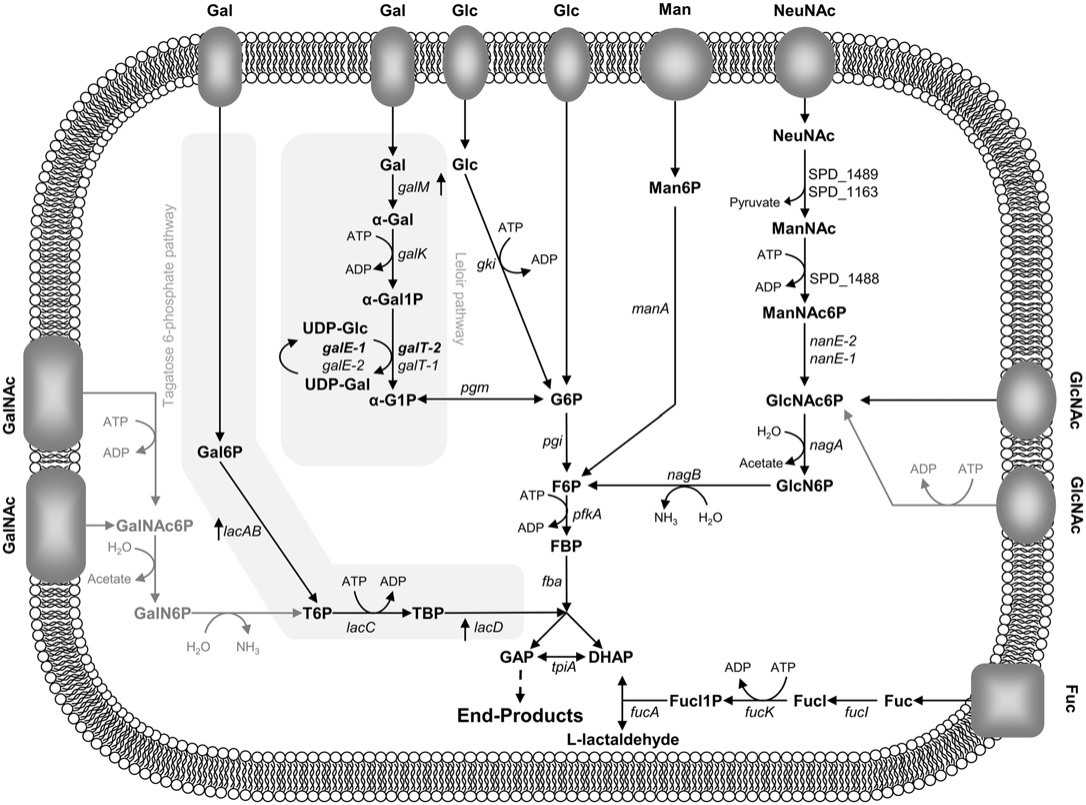
Schematic representation of the proposed pathways for the dissimilation of monosaccharides originating from deglycosylation of host glycans in *S*. *pneumoniae* D39. Pathways were reconstructed resorting to metabolic databases (MetaCyc and KEGG), genome annotations at NCBI and literature. Genes and intermediates involved in the galactose (*Gal*), mannose (*Man*), N-acetylneuraminic acid (*NeuNAc*), N-acetylgalactosamine (*GalNAc*), N-acetylglucosamine (*GlcNAc*) and fucose (*Fuc*) catabolism are shown. Initial steps of glycolysis are depicted. *galM*, aldolase 1-epimerase; *galK*, galactokinase; *galT-1*, *galT-2*, galactose 1-phosphate uridylyltransferase; *galE-1*, *galE-2*, UDP-glucose 4-epimerase; *pgm*, phosphoglucomutase/phosphomannomutase family protein; *lacA*, galactose 6-phosphate isomerase subunit LacA; *lacB*, galactose 6-phosphate isomerase subunit LacB; *lacC*, tagatose 6-phosphate kinase; *lacD*, tagatose 1,6-diphosphate aldolase; *manA*, mannose 6-phosphate isomerase; *SPD_1489*, *SPD_1163*, N-acetylneuraminate lyase; *SPD_1488*, ROK family protein, *nanE-1*, *nanE-2*, N-acetylmannosamine 6-phosphate 2-epimerase; *nagA*, N-acetylglucosamine 6-phosphate deacetylase; *nagB*, glucosamine 6-phosphate isomerase; *fucI*, L-fucose isomerase; *fucK*, L-fuculose kinase; *fucA*, L-fuculose phosphate aldolase. *α-Gal*, α-galactose; *α-Gal1P*, α-galactose 1-phosphate; *α-G1P*, α-glucose 1-phosphate; *UDP-Glc*, UDP-glucose; *UDP-Gal*, UDP-galactose; *Gal6P*, galactose 6-phosphate; *T6P*, tagatose 6-phosphate; *TBP*, tagatose 1,6-diphosphate; *Man6P*, mannose 6-phosphate; *ManNAc*, N-acetylmannosamine; *ManNAc6P*, N-acetylmannosamine 6-phosphate; *GlcNAc6P*, N-acetylglucosamine 6-phosphate; *GlcN6P*, glucosamine 6-phosphate; *Fucl*, fuculose; *Fucl1P*, fuculose 1-phosphate; *GalNAc6P*, N-acetylgalactosamine 6-phosphate; *GalN6P*, galactosamine 6-phosphate. The upper glycolytic intermediates and gene annotations are as follows: *G6P*, glucose 6-phosphate; *F6P*, fructose 6-phosphate; *FBP*, fructose 1,6-biphosphate; *GAP*, glyceraldehyde 3-phosphate; *DHAP*, dihydroxyacetone phosphate. *gki*, glucokinase; *pgi*, glucose 6-phosphate isomerase; *pfkA*, 6-phosphofructokinase; *fba*, fructose-biphosphate aldolase; *tpiA*, triosephosphate isomerase. The lower glycolytic pathway is represented by a dashed arrow. Pathways or steps present in other organisms but uncertain in D39 are represented in grey. Vertical arrows near the gene name indicate the upregulation during growth on mucin as compared to Glc, in *S*. *pneumoniae* D39.

Host glycans are rich in the carbohydrate monomers Gal, GalNAc, GlcNAc, NeuNAc, mannose (Man) and Fuc. We set out to uncover the genomic potential of *S*. *pneumoniae* D39 for utilization of these sugars and amino sugars by performing pathway reconstruction using data from the literature and deposited in metabolic databases (MetaCyc and Kegg), as well as by protein homology (BlastP) to functionally characterized enzymes (Fig 1 and S4 Table). In general, our systematic analysis confirmed genome annotations, and a schematic representation of the inferred sugar catabolic pathways is depicted in Fig 1. A more detailed description is provided as supplemental material (S4 Table and S2 Text).

Galactose can be metabolized via the Leloir or tagatose 6-phosphate (T6P) pathways (Fig 1), and homologues of the genes involved in both pathways are present in the genome (S4 Table). A duplication event of the Leloir genes, *galT* and *galE*, seems to have occurred (S4 Table), but whether the proteins are functional is unknown. Mannose is, most likely, taken up via a PTS [8] and the phosphorylated product isomerised to fructose 6-phosphate (F6P) via mannose 6-phosphate isomerase. Complete pathways for utilization of GalNAc and Fuc could not be successfully reconstituted using the tools in this work. *S*. *pneumoniae* D39 also possesses homologues of all proteins involved in the bacterial superpathway for the dissimilation of the amino sugars N-acetylneuraminate and GlcNAc (S4 Table and S2 Text) [49].

### The ability of host glycan-derived sugars to support growth is sugar dependent

**Fig 2:**
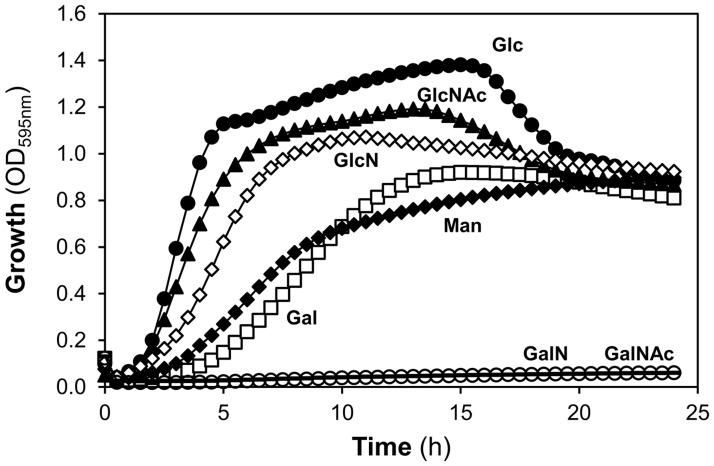
Growth of *S*. *pneumoniae* D39 in glycan-derived sugars. Growth profiles of D39 grown in CDM supplemented with 30 mM of galactose (Gal), mannose (Man), N-acetylglucosamine (GlcNAc), glucosamine (GlcN), N-acetylgalactosamine (GalNAc), galactosamine (GalN) and glucose (Glc). Growth experiments were performed at 37ºC and at an initial of pH 6.5, using a 96-well microtiter plate reader. Symbols: (closed circle) Glc; (closed triangle) GlcNAc; (open diamond) GlcN; (open square) Gal; (closed diamond) Man, (dash) GalNAc and (open circle) GalN. Growth curves are plotted in decimal scale to assess for significant differences in the growth profiles.

The presence of the genes for a full metabolic pathway in the genome does not confirm that the pathway is functioning. Thus, we assessed the ability of monosaccharide constituents of host glycans to support growth of *S*. *pneumoniae* D39 in a chemically defined medium (Fig 2). Of the monosaccharides tested, growth was observed on glucosamine, GlcNAc, Gal and Man. In contrast, *S*. *pneumoniae* was unable to use GalNAc, galactosamine (Fig 2) and NeuNAc (data not shown) as single carbon sources for growth. Fucose was not tested, since inability to grow in this sugar has been previously documented [8,50–52]. The ability of each sugar to sustain growth was consistent with the conclusions from genome analysis.

### Mucin induces expression of genes involved in utilization of Gal, GlcNAc and Man

Mucins are the most abundant glycoproteins in the human respiratory tract, and *S*. *pneumoniae* is capable of growing on mucin as sole carbon source [12]. To reveal prevalent pathways during growth on mucin, we performed a whole transcriptome analysis comparing the mRNA levels of *S*. *pneumoniae* D39 cells grown on porcine gastric mucin to those of cells grown on Glc (see Materials and Methods). The assumption for this experimental design was that genes potentially involved in the utilization of sugar moieties in mucin would be upregulated. In line with this hypothesis, 39 out of 83 genes that were significantly differentially expressed (according to the established criterion) encode for proteins with predicted functions in sugar processing (modification, uptake and catabolism) (Table 1).

**Table 1:** Expression levels of genes upregulated in *S*. *pneumoniae* D39 cells grown in mucin as compared to glucose-grown cells ^a^ ^,^ ^b^.

Locus-tag	Gene	Function	Overexpression fold
SPD_0562	*bgaA*	Beta-galactosidase	71.8
SPD_1057		PTS system transporter subunit IIB	61.3
SPD_0561		PTS system transporter subunit IIC	45.5
SPD_0068		PTS system transporter subunit IID	25.4
SPD_1053	*lacA*	Galactose 6-phosphate isomerase subunit LacA	24.5
SPD_0069		PTS system transporter subunit IIA	23.9
SPD_1052	*lacB*	Galactose 6-phosphate isomerase subunit LacB	21.7
SPD_0071	*galM*	Aldose 1-epimerase	19.4
SPD_1050	*lacD*	Tagatose 1,6-diphosphate aldolase	17.6
SPD_0559		PTS system transporter subunit IIA	15.1
SPD_0560		PTS system transporter subunit IIB	12.1
SPD_1494		Sugar ABC transporter permease	10.3
SPD_0070	*agaS*	Sugar isomerase	9.8
SPD_0063	*strH*	Beta-N-acetylhexosaminidase	8.7
SPD_1663	*treC*	Alpha,alpha-phosphotrehalase	8.0
SPD_0066		PTS system transporter subunit IIB	7.8
SPD_0287		Hyaluronate lyase	7.7
SPD_1495		Sugar ABC transporter sugar-binding protein	6.7
SPD_0293		PTS system transporter subunit IIA	6.5
SPD_1834		Bifunctional acetaldehyde-CoA/alcohol dehydrogenase	5.8
SPD_0292		Gluconate 5-dehydrogenase	5.3
SPD_1409		Sugar ABC transporter ATP-binding protein	4.8
SPD_1934	*malX*	Maltose/maltodextrin ABC transporter maltose/maltodextrin-binding protein	4.8
SPD_0297		PTS system transporter subunit IID	4.4
SPD_1496		PTS system transporter subunit IIBC	4.4
SPD_1006	*glgC*	Glucose 1-phosphate adenylyltransferase	4.4
SPD_0621	*lctO*	Lactate oxidase	4.0
SPD_1935	*malC*	Maltodextrin ABC transporter permease	3.9
SPD_0420	*pflB*	Formate acetyltransferase	3.7
SPD_1007	*glgD*	Glucose 1-phosphate adenylyltransferase, GlgD subunit	3.5
SPD_1005	*glgB*	Glycogen branching protein	2.9
SPD_1936	*malD*	Maltodextrin ABC transporter permease	2.9
SPD_0250		Pullulanase, extracellular	2.9
SPD_1989		PTS system transporter subunit IID	2.6
SPD_0427	*lacG-1*	6-phospho-beta-galactosidase	2.5
SPD_1675	*rafG*	Sugar ABC transporter permease	2.4
SPD_0925		Hydrolase	2.4
SPD_1937	*malA*	Maltodextrose utilization protein MalA	2.3
SPD_0424		PTS system cellobiose-specific transporter subunit IIC	2.2

^a^ Only genes potentially involved in sugar hydrolysis, uptake and metabolism are shown. Hypothetical proteins have been omitted.

^b^ For a complete appreciation a full list of the significantly differentially expressed genes is provided in S5 Table.

Seven of the upregulated genes were involved in the hydrolysis of sugars (Table 1). Of note, *bgaA* encoding a β-galactosidase, the activity of which results in free Gal, showed the highest differential expression value. *strH*, which codes for a *β-N*-acetylhexosaminidase, involved in the hydrolysis of terminal non-reducing *N*-acetyl-D-hexosamine residues, was the second most upregulated glycosidase (Table 1).

Among the genes implicated in sugar processing (Table 1), 49% encode components of PTS or ABC transporters. Three genes encoding a complete PTS in the galactitol family, and presumably involved in Gal uptake [8,53], showed a high positive response to mucin. In the genome of D39, this Gat-PTS transport system is located upstream of the *bgaA* locus. Also, highly overexpressed was the Man-PTS family transporter (SPD_0066-8-9) presumably involved in Gal and Man transport (Table 1 and S4 Table) [8]. In fact, genes putatively involved in Man uptake represented the highest fraction of transport systems upregulated in the presence of mucin (Table 1 and S4 Table). Moreover, a gene (SPD_1496) presumed to be involved in the translocation of GlcNAc was found differentially overexpressed (Table 1 and S4 Table), in accordance with the observed upregulation of *strH*. Mucin also induced the expression of genes potentially involved in NeuNAc uptake (Table 1 and S4 Table).

Finally, genes involved in both Gal catabolic routes, *lacAB* and *lacD* of T6P pathway and *galM* of the Leloir pathway were highly overexpressed during growth on mucin (Table 1 and Fig 1). Expression levels of selected genes, obtained by qRT-PCR, confirmed the transcriptome results (S6 Table).

In summary, our transcriptome analysis revealed that mucin induced the expression of genes required to benefit from Gal, GlcNAc and Man residues present in host glycans. Capitalizing on the growth and expression data, we surmised that these sugars are important carbon sources for D39 during colonisation of the nasopharynx. Hence, we set out to characterize growth and validate predicted metabolic pathways for utilization of Gal, GlcNAc and Man.

### Growth properties on Gal, GlcNAc and Man or on a mixture thereof

Growth parameters and fermentation end-products were determined in batch cultures of *S*. *pneumoniae* D39 using chemically defined medium and two concentrations of Gal, GlcNAc or Man: 13±1 mM and a higher non-limiting 34±2 mM.

#### Growth profiles on Gal, Man and GlcNAc

**Fig 3:**
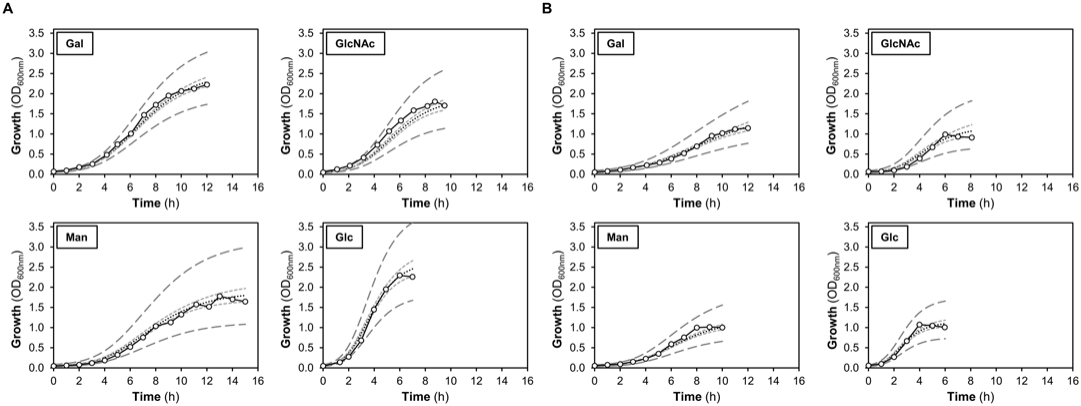
Representative growth profiles of *S*. *pneumoniae* D39 grown in CDM in the presence of two different concentrations of galactose (Gal), N-acetylglucosamine (GlcNAc), mannose (Man) or glucose (Glc). Growth profiles in 80 ml batch cultures using (*A*) excess substrate concentration (34±2 mM) and (*B*) a 3-fold lower initial substrate concentration (13±1 mM). Representative growth curves (empty dots) were selected based on the established criterion (see Materials and Methods). The experimental data was fitted using the Gompertz model with all replicates (estimated curve in black dashed line) and the 95% confidence bands (small dashed light grey lines) and 95% predicted bands (big dashed dark grey lines) were determined (see Materials and Methods). Growth was performed at 37ºC, under semi-anaerobic conditions, without pH control (initial pH 6.5). Growth curves are plotted in decimal scale to allow the visual discernment between significantly differences in the growth parameters using the two initial substrate concentrations.

Representative growth profiles (experimental data) and model 95% confidence and prediction curves that best describe the growth data are depicted in Fig 3. In general, the model fits well with the experimental data. The 95% confidence curves (Fig 3) reveal that the larger differences between biological growth replicas occur at the final stage of the growth, as shown by higher discrepancies in the final optical density (OD_max_) rather than specific growth rate (μ_max_). The parameters derived from the growth analysis are shown in Table 2. Analysis through ANOVA and pairwise t-tests allowed assessment of the statistical significance of the growth parameter differences across the experimental conditions (S2 Table).

**Table 2:** Growth and energetic parameters of *S*. *pneumoniae* D39 on sugars present in host glycans.

	GlcNAc		Gal		Man		Glc		Mix
**[substrate]_initial_ (mM)**	**30.8±0.6**	**12.3±0.6**	**33.7±1.5**	**13.4±1.3**	**33.4±0.3**	**12.4±0.1**	**34.2±1.8**	**12.9±0.4**	**6.5±0.3** ^a^
**µmax (h^-1^)**	0.55±0.06	0.54±0.08	0.48±0.04	0.32±0.03	0.45±0.04	0.44±0.04	0.93±0.07	0.93±0.09	0.61±0.05 0.07±0.01^b^
**OD_600max_**	1.76±0.25	0.99±0.08	2.16±0.12	1.12±0.26	1.77±0.16	1.03±0.08	2.29±0.17	1.06±0.04	1.19±0.11
**ΔpH**	1.10±0.08	0.41±0.05	1.02±0.06	0.48±0.07	1.23±0.15	0.44±0.03	1.28±0.09	0.44±0.03	0.57±0.08
**Substrate Consumed** (%)	93±6	100±0	69±9	84±7	87±10	100±0	92±4	100±0	86±2
**Substrate Recovery**	87±5	84±3	72±1	76±9	85±9	89±3	86±5	84±2	79±3
**Redox Balance**	86±5	83±3	70±2	75±5	85±9	85±3	85±5	82±1	81±4
**ATP yield (mol mol^-1^ substrate)**	1.77±0.09	1.74±0.08	2.08±0.03	2.27±0.31	1.80±0.21	1.98±0.04	1.75±0.11	1.71±0.04	1.73±0.06
**YATP (g biomass mol^-1^ATP)**	12.1±0.7	16.8±0.9	17.5±1.4	14.9±0.5	13.6±1.4	16.2±0.4	15.3±0.8	18.8±0.0	16.4±0.4
**Ybiomass (g mol^-1^ substrate)**	21.4±2.4	29.3±2.9	36.3±2.4	34.1±5.8	24.3±0.3	31.9±0.1	26.7±0.3	32.0±0.8	28.3±1.8

Growth rate, maximal biomass, yields, carbon and redox balances, substrate consumed and variation of pH were obtained for *S*. *pneumoniae* D39 grown in CDM in the presence of N-acetylglucosamine (GlcNAc), galactose (Gal), mannose (Man) and glucose (Glc) using two substrate concentrations or in a mixture of Gal, Man and GlcNAc (*circa* 6.5 mM each). Growth was performed at 37ºC without pH control (initial pH 6.5). Values represent the average and standard deviation of at least two independent growth experiments and are estimated at the time point of growth arrest (maximal biomass).

^a^The value represents the average ± standard deviation of the concentration of sugars present in the mixture. Individual values are: Gal, 6.4±0.7 mM; Man, 6.6±0.1 mM and GlcNAc, 6.7±0.2 mM.

^b^Second specific growth rate.

*ΔpH* is the difference between the initial pH and the pH at the time of growth arrest; *substrate recovery* is the percentage of carbon in metabolized sugar that is recovered in the fermentation products (lactate, ethanol, acetate, and formate); *redox balance* is the ratio between [lactate]+2×[ethanol] and 2×[substrate consumed] multiplied by 100.

In medium containing the higher substrate concentration, GlcNAc supported a significantly faster growth than Gal and Man (Table 2 and S2 Table). However, no significant differences (*p* = 0.15) were found between cells growing on higher concentrations of Gal or Man, with the specific growth rate similar in both conditions (Table 2 and S2 Table). In contrast, for the lower substrate concentration, the growth rate was sugar dependent with the amino sugar supporting a significantly higher growth rate than Man or Gal (Table 2 and S2 Table). The specific growth rate is independent of the initial substrate concentration (Table 2), except for Gal which supports higher growth rates when the substrate is in excess. The involvement of a low affinity transporter in the uptake of this sugar would explain this behaviour.

When the higher substrate concentration was used, Gal supported the highest final biomass, which was similar to that on Glc (*p* = 0.06) (Table 2 and S2 Table). No significant differences in the final biomass (*p* = 0.89) were observed between growths on GlcNAc and Man, which were lower than that on Gal (Table 2 and S2 Table). At the lower substrate concentration the biomass formed was similar for the glycan-derived sugars and Glc, and unsurprisingly lower than those obtained using non-limiting sugar concentrations (Fig 3, Table 2 and S2 Table).

In medium with Gal, growth arrest occurred before substrate depletion, even for the low Gal concentration (Table 2). For the higher sugar concentrations, a pH decrease of about one unit was observed at the onset of stationary phase, which is consistent with growth arrest due to acidification. However, the observed change in pH of 0.5 units does not explain the arrest of growth in the lower Gal concentration. Even though a full explanation cannot be put forward, the possession of only low-affinity Gal importers can be proposed as a cause for growth slow-down with decreasing Gal concentrations.

#### Fermentation products

End-products resulting from the fermentations of Gal, GlcNAc and Man are shown in Table 3. S. pneumoniae displayed a fully homolactic fermentation profile when GlcNAc was the sole carbon source, regardless of the initial concentration. In addition to lactate, acetate, ethanol and formate were produced as minor fermentation products (Table 3). On Man, the fermentation profile was still mainly homolactic. However, a shift towards mixed acid fermentation was evident, accounting for 9% and 17% of the substrate consumed in the higher and lower substrate concentrations, respectively. In contrast, cells grown on Gal showed a pronounced mixed acid fermentation (Table 3), independently of the initial concentration of sugar. Formate, ethanol and acetate were produced in the ratio 2:1:1, as expected from mixed acid fermentation under anaerobic conditions. Lactate was detected as a minor fermentation product, accounting for 8% and 2% of the consumed Gal, for the higher and lower substrate concentration, respectively (Table 3). The shift towards mixed acid fermentation profile was generally higher for the lower substrate concentrations.

**Table 3:** End-products derived from the catabolism of N-acetylglucosamine (GlcNAc), galactose (Gal), mannose (Man) and glucose (Glc) by S. pneumoniae D39, using 34±2 mM or 13±1 mM initial substrate concentrations.

	Higher substrate concentration				Lower substrate concentration			
Sugar	GlcNAc	Man	Gal	Glc	GlcNAc	Man	Gal	Glc
**[End-products] (mM)**								
**Lactate**	49.02±8.19	43.80±0.11	3.88±0.54	53.10±1.59	19.42±0.35	17.27±1.63	0.50±0.00	20.93±0.29
**Acetate**	0.92±0.10	2.68±0.17	14.91±1.24	0.83±0.38	0.79±0.14	2.44±0.26	8.43±0.06	0.46±0.12
**Ethanol**	0.48±0.08	2.66±1.67	14.30±1.53	0.10±0.04	0.42±0.11	1.64±0.43	8.16±0.86	0.08±0.02
**Formate**	1.62±0.43	5.10±0.75	29.43±1.85	BDL	1.82±0.73	3.77±0.44	16.53±0.84	BDL

Growth was done in CDM supplemented with the appropriate sugar, at 37ºC, under semi-anaerobic conditions, without pH control (initial pH 6.5). The results represent averages of at least two experiments and the error bars the standard deviation.

BDL, below detection limit. In glucose grown cells, formate was produced, but in quantities below the limit of quantification.

The calculated values for substrate recovery are in good agreement with fermentative metabolism (above 80%). On Gal, carbon balances in the 70% range were determined, indicating an additional carbon sink (Table 2).

#### Growth profiles, substrate consumption and end-products of fermentation in a mixture of Gal, GlcNAc and Man

**Fig 4:**
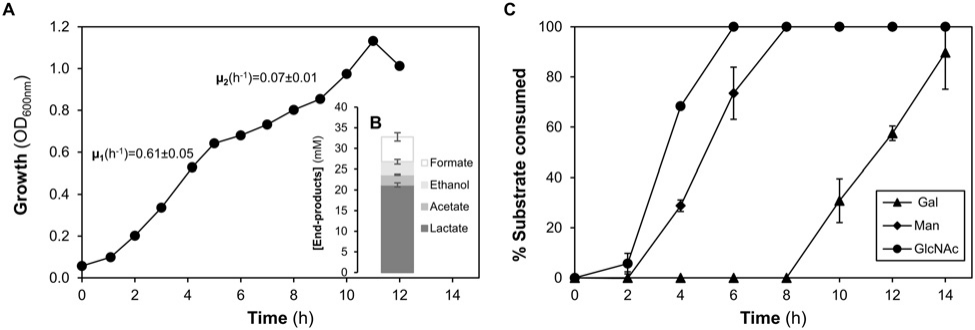
Growth profile of D39 in a mixture of galactose (Gal), mannose (Man) and N-acetylglucosamine (GlcNAc). (*A*) Representative growth curve of *S*. *pneumoniae* D39 grown in CDM supplemented with a mixture of Gal, GlcNAc and Man (6.5 mM each), at 37ºC, under semi-anaerobic conditions, without pH control (initial pH 6.5). (*B*) End-products of fermentation derived from the catabolism of the sugars in the mixture (Gal, Man and GlcNAc), determined at the time of growth arrest (maximal biomass). (*C*) Gal, Man and GlcNAc consumption, over time, during the growth of *S*. *pneumoniae* D39 in CDM. The values are percentages of the initial sugar concentration for each sugar. The representative growth curve was selected based on the established criterion (see Materials and Methods). The results represent averages of two independent growths and the error bars represent the standard deviation.

In its ecological niche, *S*. *pneumoniae* is exposed to a multiplicity of sugars. Thus, we set out to evaluate growth on a sugar mixture containing Gal, GlcNAc and Man. An initial concentration of approximately 6.5 mM for each carbohydrate, was tested (Fig 4). On the sugar mixture, *S*. *pneumoniae* D39 displayed a biphasic growth profile. The maximal growth rate (μ_1_) was observed within the first 4 h of growth and was about nine times higher than the second growth rate (μ_2_) (Fig 4A). Interestingly, μ_1_ was similar to that determined in the presence of the lower concentration of GlcNAc alone (Table 2), suggesting that this sugar was consumed first.

The profile of sugar utilization was determined by measuring the sugars in the culture medium using ^1^H-NMR (Fig 4C). We confirmed that GlcNAc was consumed first and was totally depleted after 6 h of growth. Consumption of Man started while GlcNAc was still available, and its depletion occurred 8 h after inoculation. Interestingly, Gal was only used after depletion of GlcNAc and Man. This is in good agreement with the two distinct growth rates found: the first is related to GlcNAc and Man consumption, whereas the second mostly reflects the utilization of Gal.

In the sugar mixture, the end-products profile was mainly homolactic, with lactate as the major product (21.2 ± 0.5 mM) (Fig 4B). Minor quantities of formate, ethanol and acetate were formed in a proportion of 2:1:1. (Fig 4B). Growth arrest was most likely not due to acidification, since only a modest change in pH (ΔpH) was registered (Table 2). On the other hand, Gal was not fully consumed, with approximately 40% remaining in the medium at the time of growth arrest (12 h after inoculation) (Table 2 and Fig 4C). A similar behaviour was observed when Gal was used as single carbon source. These results demonstrated that *S*. *pneumoniae* D39 is able to metabolize different carbon sources simultaneously or sequentially. In a mixture consisting of 6.5 mM GlcNAc, Man and Gal, strain D39 had a preference for GlcNAc, but could use Man concurrently. Gal was the least preferred sugar, and was only consumed after exhaustion of the two other carbon sources.

### Catabolic pathways for the utilization of Gal, GlcNAc and Man as assessed using biochemical and molecular tools

Experimental confirmation of the predicted metabolic routes for the catabolism of Gal, Man and GlcNAc was performed at the biochemical level through metabolite profiling by ^31^P-NMR and enzyme activity measurements, and at the genetic level by mutating key genes in the pathways.

#### Intracellular metabolites during growth on glycan-derived sugars

**Fig 5:**
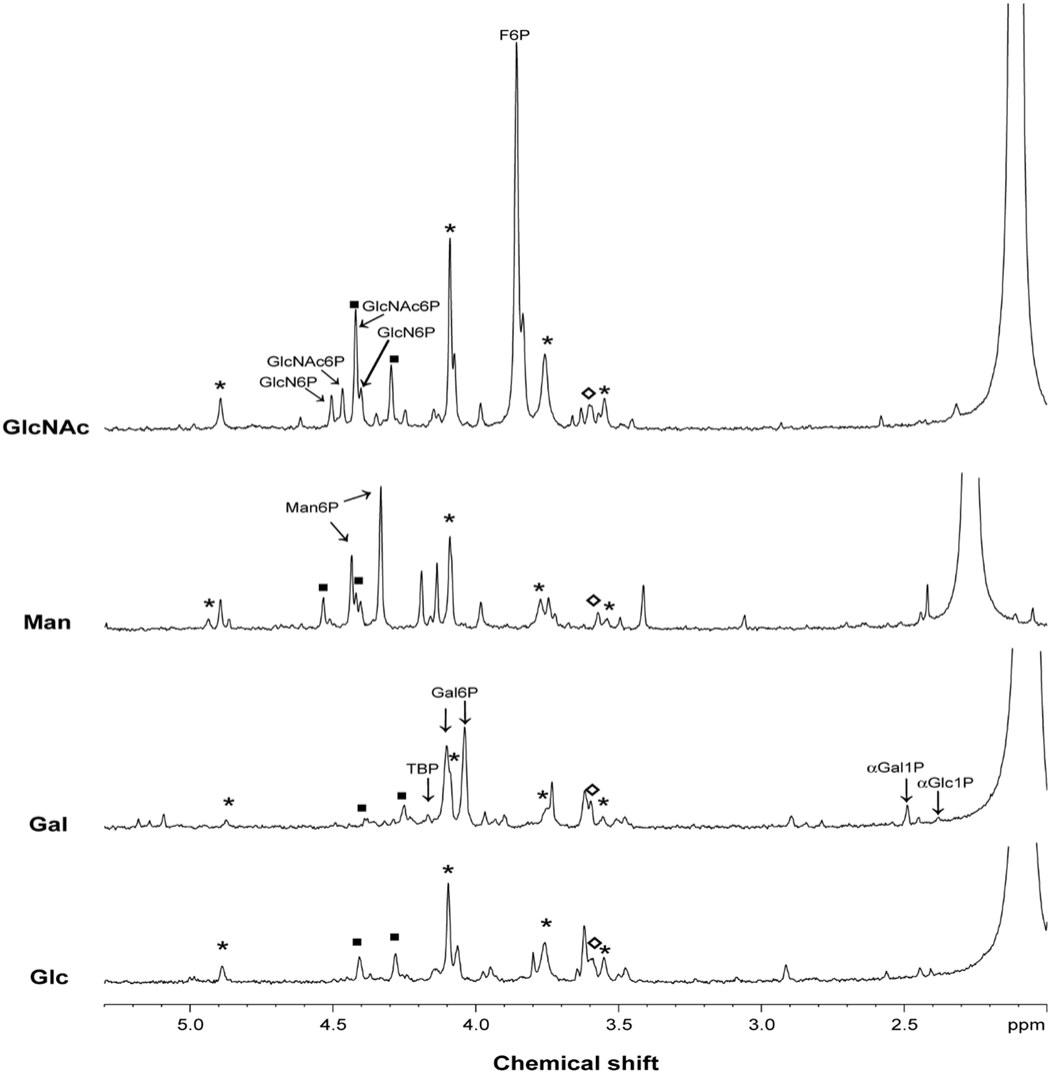
Phosphomonoester region of ^31^P-NMR spectra of ethanol extracts obtained in late-exponential cells of *S*. *pneumoniae* D39 grown in CDM with galactose (Gal), mannose (Man) or N-acetylglucosamine (GlcNAc). Ethanol extracts of glucose (Glc) grown cells were used as control. The glycolytic intermediates fructose 1,6-biphosphate (FBP), glucose 6-phosphate (G6P) and 3-phosphoglycerate (3-PGA) were firmly assigned. Abbreviations: αGal1P, α-galactose 1-phosphate; αGlc1P, α-glucose 1-phosphate; TBP, tagatose 1,6-diphosphate; Gal6P, galactose 6-phosphate; Man6P, mannose 6-phosphate; GlcN6P, glucosamine 6-phosphate; GlcNAc6P, N-acetylglucosamine 6-phosphate and F6P, fructose 6-phosphate. Identification of phosphorylated intermediates was performed by spiking the ethanol extracts with pure compounds. Symbols: (asterisk) FBP, (closed square) G6P, (open diamond) 3-PGA.

Ethanol extracts of growing cells were examined for phosphorylated intermediates of catabolic pathways by targeted metabolomics using 31P-NMR. In extracts derived from S. pneumoniae D39 Gal-grown cells, phosphorylated intermediates of the Leloir pathway, α-galactose 1-phosphate (α-Gal1P) and α-glucose 1-phosphate (α-Glc1P), as well as phosphorylated metabolites involved in the T6P pathway: galactose 6-phosphate (Gal6P) and tagatose 1,6-diphosphate (TBP), were detected (Fig 5), indicating the active presence of both catabolic routes for Gal catabolism. These data are in agreement with an earlier report for a different isolate of S. pneumoniae strain D39 [9]. The accumulation of mannose 6-phosphate (Man6P) during growth on Man is a strong indication of the functioning of the predicted catabolic route (Figs 1 and 5). The intracellular intermediates predicted in the catabolic pathway of GlcNAc, N-acetylglucosamine 6-phosphate (GlcNAc6P) and glucosamine 6-phosphate (GlcN6P), were detected in the extracts of cells grown on this carbon source (Figs 1 and 5). Surprisingly, fructose 6-phosphate (F6P) was highly accumulated under this condition.

Thus, the occurrence of specific phosphorylated metabolites in cell extracts correlated well with the predicted metabolic intermediates in the catabolism of each sugar.

#### Enzymatic activities of key enzymes involved in the catabolism of glycan-derived sugars

To further substantiate the functioning of the predicted pathways, we selected enzymes presumed to be required for pathway activity, galactokinase (GalK, SPD_1634) and tagatose 1,6-diphosphate aldolase (LacD, SPD_1050) for Gal, mannose 6-phosphate isomerase (ManA, SPD_0641) for Man and N-acetylglucosamine 6-phosphate deacetylase (NagA, SPD_1866) for GlcNAc, and determined their specific activities using specific biochemical assays (see Materials and Methods).

On galactose, the specific activities of GalK and LacD in cell-free extracts of D39 were considerably higher than those measured on Glc-grown cells (Table 4), suggesting induction of the pathway on Gal. These data are consistent with the functioning of both pathways during growth on Gal. The GalK specific activity was higher than the LacD specific activity, under the conditions assayed (Table 4).

**Table 4:** Enzyme specific activities determined in extracts of *S*. *pneumoniae* derived from cells grown to late-exponential phase of growth in CDM supplemented with different monosaccharides.

		Specific activities (U mg protein-1)			
Strain	Growth condition (carbon source)	GalK	LacD	NagA	ManA
D39	Gal	0.89 ± 0.11	0.08 ± 0.01	-	-
D39	GlcNAc	-	-	0.04 ± 0.00	-
D39	Man	-	-	-	0.10 ± 0.01
D39	Glc	BDL	BDL	0.02 ± 0.00	0.07 ± 0.01
D39Δ*galK*	Glc	BDL	-	-	-
D39Δ*lacD*	Gal	-	BDL	-	-
D39Δ*nagA*	Glc	-	-	BDL	-
D39Δ*manA*	Glc	-	-	-	0.04 ± 0.01

Specific activity is expressed as units (U) (μmol min^-1^) per milligram of protein (U mg protein^-1^).

GalK specific activity is defined as the amount of protein required for the formation of 1 μmol of Gal1P min^-1^ mg protein^-1^. LacD specific activity is given as the amount of protein required to catalyse the oxidation of 1 μmol of NADH min^-1^ mg protein^-1^. NagA and ManA specific activities are the amount of protein to reduce 1 μmol of NADP min^-1^ mg protein^-1^. The values reported represent averages ± standard deviation obtained in cell-free extracts of at least two independent cultures.

^-^, not determined.

BDL, below detection limit.

The specific activity of N-acetylglucosamine 6-phosphate deacetylase (NagA), the enzyme dedicated to GlcNAc catabolism, was 2-fold higher when grown on GlcNAc than in Glc-grown cells (Table 4). The method to assay NagA, couples its activity to that of glucosamine 6-phosphate isomerase (NagB). The latter was not limiting in the assay, since its specific activity was 4 times higher than that of NagA (data not shown).

The key enzyme of the Man catabolic pathway, ManA, was detected in Man-grown cells, but the level was only marginally reduced (30%) in Glc-grown cells (Table 4).

In summary, enzyme activities of dedicated sugar catabolic enzymes were detected for each metabolic pathway assayed, indicating the operability of the dissimilation routes proposed.

#### Genetic confirmation of pathway functionality

**Fig 6:**
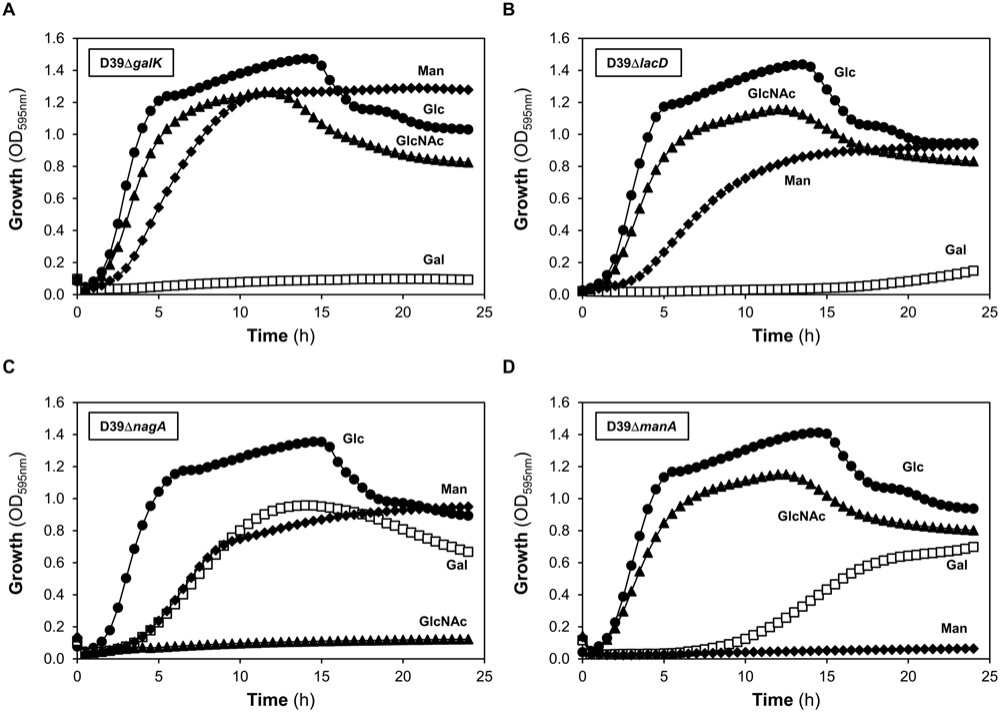
Growth profile of D39 sugar-specific mutants. Growth phenotypes of (*A*) D39Δ*galK*, (*B*) D39Δ*lacD*, (*C*) D39Δ*nagA* and (*D*) D39Δ*manA*. Growth was performed in CDM supplemented with galactose (Gal), N-acetylglucosamine (GlcNAc), mannose (Man) or glucose (Glc), using the 96-well microplate reader. Symbols: (closed triangle) GlcNAc; (closed diamond) Man; (open square) Gal and (closed circle) Glc.

The biochemical approach to investigate the Gal, Man and GlcNAc catabolic pathways was complemented by a genetic approach. Mutants in key enzymatic steps of each catabolic route were constructed by allelic replacement mutagenesis, yielding D39ΔgalK, D39ΔlacD, D39ΔmanA and D39ΔnagA (S3 Fig). The mutations were confirmed by growth profiles and enzyme activity measurement (Table 4 and Fig 6). The deletion mutants lost the activity encoded by the inactivated gene, except for the D39ΔmanA mutant (Table 4). In Glc-grown D39ΔmanA, the activity of mannose 6-phosphate isomerase showed a 43% and 60% reduction compared to that in Glc- or Man-grown wild type D39 cells, respectively (Table 4).

Importantly, the D39Δ*manA* or D39Δ*nagA* strains were unable to use Man or GlcNAc as sole carbon source, respectively (Fig 6C and 6D). D39Δ*lacD* was able to grow on Gal, although only after a lag period of *circa* 20 h (Fig 6B). A mutant in both Gal catabolic pathways was constructed, D39Δ*lacD*Δ*galK*, which lost the ability to grow on this monosaccharide. Unexpectedly, inactivation of *galK* alone resulted in a strain unable to grow on Gal, even though the T6P pathway was still intact (Fig 6A). All pathway-specific mutants were able to grow on sugars other than the substrate of the targeted catabolic pathway (Fig 6). To ensure that growth abrogation was unrelated to possible polar effects, complementation studies were conducted (see Materials and Methods, S4 Fig). D39Δ*manA and* D39Δ*lacD* complemented with *manA* and *lacD* under the Zn^2+^-inducible promoter (P_czcD_) [54], had fully restored growth on Man and Gal, respectively. It is worth noting that growth on Man was better in the complemented D39Δ*manA* as compared to wild type D39, suggesting that the ManA activity level might be a limiting factor for Man utilization (S4D Fig). For the D39Δ*nagA*, the ability to grow on GlcNAc was recovered by expressing, in *trans*, *nagA* under its own promoter (S4C Fig).

For D39Δ*galK*, the ability to grow on Gal was not recovered by complementation in *trans* with *galK*. RNA-Seq data [55] showed that *galK* is co-transcribed with the downstream gene *galT-2* and a polar effect of the *galK* deletion on *galT-2* is therefore possible (S3 Fig). Indeed, the growth phenotype on Gal could be restored by complementation in *trans* with *galKgalT-2* (S4A Fig). Importantly, complementation with *galT-2* alone was not successful, suggesting that abrogation of growth on Gal is a consequence of *galK* inactivation.

To further analyse the effect of gene deletions in the Leloir pathway, a D39Δ*galT-2* mutant was constructed. The D39Δ*galT-2* grew on Gal, but the time to reach maximal biomass was 2.3-fold longer as compared to the wild type (data not shown). This result indicates the occurrence of an alternative galactose 1-phosphate activity, likely to be encoded by *galT-1*. On Gal, the expression level of *galT-1* in D39Δ*galT-2* was 49-fold higher than in D39, as shown by qRT-PCR (S7 Table). Expression of *lacD* was 87-fold higher in Gal-grown D39Δ*galT-2* (S7 Table) than in Gal-grown wild type, indicating that in the D39Δ*galT-2* mutant the *lac* operon is expressed and the T6P pathway is active.

### Attenuated virulence in the absence of a functional Gal pathway

The contribution of genes encoding proteins involved in catabolism of host-derived glycans was tested in mouse models of colonisation, and of models of bronchopneumonia with bacteraemia that result from intranasal infection. While the bronchopneumonia model allows evaluation of factors that are important for acute infection and invasiveness, the colonisation model is ideal to evaluate the determinants of longer term pneumococcal survival *in vivo* [56].

**Fig 7:**
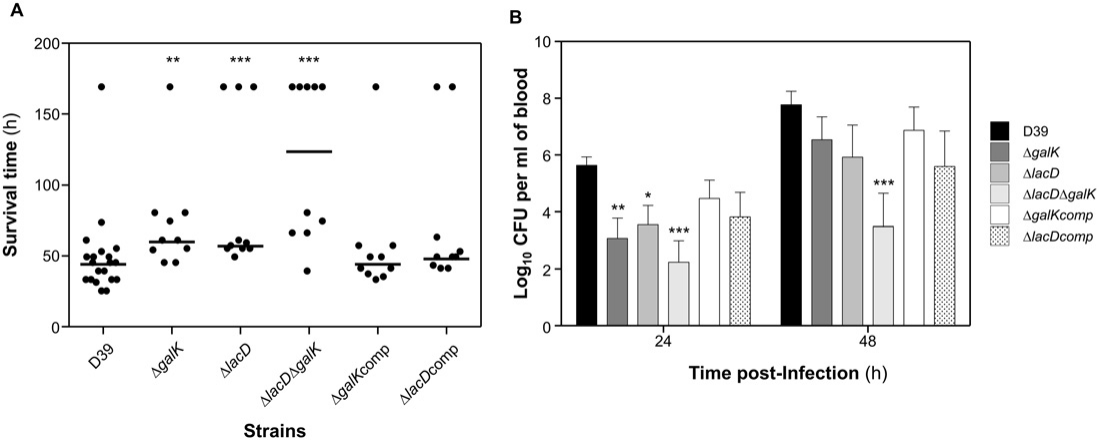
Impaired virulence of pneumococcal strains defective in galactose catabolic pathways following intranasal infection. (*A*) Survival time of mice after infection with approximately 1 X 10^6^ CFU pneumococci. Symbols show the times mice became severely lethargic. The horizontal bars mark the median times to the severely lethargic state. (*B*) Growth of bacteria in the blood. Each point is the mean of data from ten mice except for D39, which represents 20 mice. Error bars show the standard error of the mean. Symbols: * *p*<0.05; ** *p*<0.01; *** *p*<0.001.

Mice infected with the mutants in Gal catabolic pathways survived significantly longer than wild type D39 strain in the bronchopneumonia model (Fig 7A) (average survival time of mice infected with: D39 44±30.7 h, n = 20; D39Δ*galK* 60±36.3 h, n = 10; D39Δ*lacD* 59±54.1 h, n = 10 and D39Δ*lacD*Δ*galK* 123±55.8 h, n = 10; *p*<0.01 for D39Δ*galK*, and p<0.001 for D39Δ*lacD* and D39Δ*lacD*Δ*galK*; Mann-Whitney U test). In addition, reintroduction of an intact copy of *galK* and *lacD* into D39Δ*galK* and D39Δ*lacD*, respectively, reconstituted the virulence of these strains with the median survival times of mice infected with D39Δ*galK*comp (44±40.3 h, n = 10) and D39Δ*lacD*comp (51.2±40.3 h, n = 10), not significantly different from the wild type infected cohort (*p*>0.05). This shows that the observed reduction in virulence was not due to polar effect of mutations.

The progression of bacteraemia in animals infected with the pneumococcal strains was also determined. At 24 h post-infection mice infected either with D39Δ*galK* (log_10_ 3.10±0.7, n = 10), D39Δ*lacD* (log_10_ 3.54±0.6, n = 10), or D39Δ*lacD*Δ*galK* (log_10_ 2.23±0.75, n = 10) had significantly lower mean CFU ml^-1^ of bacteria in the blood than that of the wild type infected cohort (log_10_ 5.21±0.4, n = 20) (*p*<0.01 for D39Δ*galK*, *p*<0.05 for D39Δ*lacD*, and *p*<0.001 for D39Δ*lacD*Δ*galK*) (Fig 7B). At 48 h post-infection, the blood bacterial counts recovered from D39Δ*lacD*Δ*galK* (log_10_ 3.48±1.16, n = 10) infected cohort was still significantly lower than that of the wild type infected (log_10_ 7.76±0.48, n = 20) (p<0.001), however, the numbers of D39Δ*galK* (log_10_ 6.53±0.81, n = 10) and D39Δ*lacD* (log_10_ 5.92±1.13, n = 10) were similar to the wild type (p>0.05). Furthermore, no significant difference could be detected in the numbers of D39Δ*galK*comp (24 h: log_10_ 4.47±0.64 and 48 h: log_10_ 6.86±0.82 n = 10), D39Δ*lacD*comp (24 h: log_10_ 3.81±0.86 and 48 h: log_10_ 5.59±1.24 n = 10), and the wild type at 24 and 48 h post-infection (*p*>0.05). D39Δ*manA* and D39Δ*nagA* were also tested in bronchopneumonia model, however, the virulence properties of these strains were similar to the wild type (data not shown).

**Fig 8:**
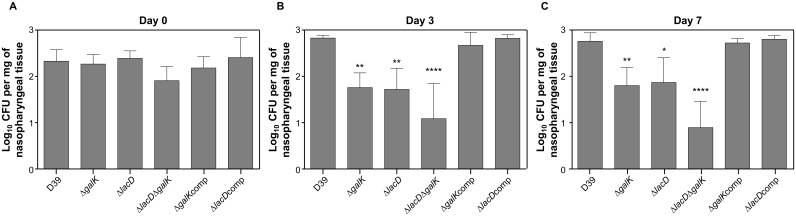
Pneumococcal strains defective in galactose catabolic pathways are less able to colonise nasopharynx. Mice were infected with approximately 1 X 10^5^ CFU pneumococci. At predetermined times, five mice were culled, and CFU mg^-1^ of bacteria were determined by serial dilutions of nasopharyngeal homogenates. Each column represents the mean of data from five mice. Error bars show the standard error of the mean. Symbols: * *p*<0.05; ** *p*<0.01, **** *p*<0.0001.

In the colonisation model, the counts for all the pneumococcal strains were determined in nasopharyngeal tissue at the time of infection, and at 3 and 7 days after infection (Fig 8). The results show that at 3 and 7 days post-infection the numbers of D39Δ*galK* (log_10_ 1.75±0.14 and log_10_ 1.80±0.17 n = 5), D39Δ*lacD* (log_10_ 1.72±0.2 and log_10_ 1.87±0.24, n = 5), and D39Δ*lacD*Δ*galK* (log_10_ 1.08±0.33 and log_10_ 0.89±0.25, n = 5) were significantly lower than the counts of wild type (log_10_ 2.82±0.02; and log_10_ 2.77±0.08, n = 5, for days 3 and 7, respectively) (*p*<0.01 for D39Δ*galK*, *p*<0.0001 for D39Δ*lacD*Δ*galK*, and *p*<0.01 and *p*<0.05 for D39Δ*lacD* for 3 and 7 days post-infection, respectively). Similar to the bronchopneumonia model, in the colonisation model no phenotypic differences were observed between the wild type and D39*ΔgalK*comp, D39 *ΔlacD*comp (Fig 8) (*p*>0.05), and the wild type and D39 *ΔmanA* and D39 *ΔnagA* (data not shown).

In addition to respiratory infection models, we also tested all the strains in a bacteraemia model through direct administration of bacteria through a tail vein in order to distinguish niche-specific contribution of individual pneumococcal proteins. There was no difference in the median survival times of cohorts (S5A Fig) (*p*>0.05). In addition, the mutants had grown as well as the wild type strain in the blood at 24 and 48 h post-infection (S5B Fig), showing that the observed reduction in virulence and colonisation in D39Δ*galK*, D39Δ*lacD* and D39Δ*lacD*Δ*galK* was specific to the respiratory tract.

Overall, the results showed that Gal catabolic mutants, particularly D39Δ*lacD*Δ*galK*, were attenuated in the ability to colonise the nasopharynx and have reduced virulence in a respiratory infection mouse model.

## Discussion and Conclusions

The study of *S*. *pneumoniae* has been heavily focused on factors that directly impinge on host-pathogen interactions, such as toxins, cell wall components, adhesins and capsule [3]. In contrast, investigation of pneumococcal physiology has only recently been addressed, in spite of it being a fundamental aspect of pneumococcal survival *in vivo*. This important pathogen is a strictly fermentative organism, which possesses one of the highest genomic abundances of genes encoding sugar transporters [6,7,24]. Thus, we surmised that the ability to take up and metabolize sugars is of key importance for the lifestyle of *S*. *pneumoniae*. Our view is strongly supported by previous studies consistently identifying genes involved in sugar catabolism as essential for virulence [11,57–61] as well as reports showing that sugar transporters contribute to *S*. *pneumoniae* colonisation and disease [16,62–64]. In the nasopharynx free sugars are scarce, but recent findings indicate that sugars derived from deglycosylation provide suitable carbon and energy sources for nasopharyngeal growth [12,14,15]. In order to understand the role of different host derived sugars in pneumococcal lifestyle and pathogenicity, we followed a top-down approach to identify, establish and validate functional sugar-specific catabolic pathways implicated in the utilization of sugars that may originate from glycan deglycosylation and evaluated the impact of sugar-specific pathways on the ability to colonise and cause disease *in vivo*.

Despite the vast diversity of host glyconjugates, their glycan portions are often composed of the monosaccharides GalNAc, Gal, NeuNAc, GlcNAc, Fuc, Man, Glc and fructose. While the first five sugars are widespread in both N- and O-glycans (*e*.*g*. mucins), the other three are generally restricted to N-glycans. *S*. *pneumoniae* can grow on mucin as sole carbon source [12]. This ability is associated to a range of glycosidases that hydrolyse the mucins releasing to the medium neuraminic acids (NeuNAc and N-glycolylneuraminic acid), Gal and GalNAc, and to a less extent also GlcNAc and Fuc [14]. From our own analysis of the genomic content and the work of others [8,9,14,39,49,63], *S*. *pneumoniae* D39 harbours genes to potentially catabolise the glycan components Gal, GlcNAc, NeuNAc and Man. A bias towards utilization of the monosaccharide Gal and GlcNAc by *S*. *pneumoniae* D39 during growth on an O-glycan was hypothesised, since the monosaccharides account respectively for 14% and 30% (wt/vol) of the mucin [14]. Furthermore, *S*. *pneumoniae* is equipped with the machinery to utilize those constituents including glycosidases (at least two galactosidases, BgaA and BgaC, and a *N*-acetylglucosaminidase StrH [14,20,65,66]), putative transporters and catabolic genes (S4 Table). This hypothesis was corroborated in a transcriptome analysis comparing gene expression of cells growing on porcine gastric mucin to Glc. Of the genes showing significant increased expression on mucin, the vast majority was implicated in the uptake and internal metabolism of Gal (25% of genes involved in sugar metabolism (Table 1)), and GlcNAc. Of note, mucin highly induced the expression of *bgaA* and *strH* encoding a β-galactosidase and a *β-N*-acetylglucosaminidase, whose activities result in free Gal and GlcNAc, respectively [18,65–68]. Growth of *S*. *pneumoniae* on mucin was shown to reduce by 30% the content in Gal of the glycoprotein, while the content in GlcNAc was not altered. This was rationalized as resulting from the complex structure of mucin, in which GlcNAc residues might be masked by other sugars.

Genes associated with the transport of Man, a monosaccharide generally found in N-glycans, were also upregulated in mucin. Like Gal and GlcNAc, Man could sustain growth of *S*. *pneumoniae* D39 in a chemically defined medium (Fig 2). Whether all the transporters upregulated show affinity for mannose remains to be investigated. Activity of α-mannosidase has been described for *S*. *pneumoniae* and homologues of previously characterized streptococcal mannosidase genes are found in the genome of D39 [69–71], but these genes were not differentially expressed in mucin-grown cells.

Previously, it was shown that growth on mucin required the activity of neuraminidase A (NanA) [12]. Initial removal of terminal NeuNAc seems to be essential for further breakdown by other glycosidases and subsequent utilization of the glycan-derived sugars [14,18,72]. In our study, neuraminidase A was not significantly induced in presence of mucin as compared to Glc, which leads us to propose that its constitutive expression is sufficient to ensure the activity level to remove terminal NeuNAc. Furthermore, we (and others) verified that NeuNAc cannot sustain growth of strain D39 in chemically defined medium, most likely due to a frame shift mutation in the N-acetylneuraminate lyase gene of the *nanAB* operon [8]. The mucin derivatives GalNAc and Fuc also failed to support growth of *S*. *pneumoniae*. For GalNAc we could not firmly identify genes encoding the activities converting N-acetylgalactosamine 6-phosphate to tagatose 6-phosphate, although genes with homology to *E*. *coli* counterparts are present in the genome (S2 Text). Genes implicated in the downstream processing of L-lactaldehyde, the product of L-fuculose phosphate aldolase in the Fuc pathway, were not found (S2 Text) [8]. This inability to identify the full catabolic pathways for Fuc and GalNAc is in good agreement with previous reports that *S*. *pneumoniae* is unable to grow on these sugars as sole carbon sources [8,50–52].

Overall, our integrated approach combining genomic, transcriptomic and growth data identified Gal, GlcNAc and Man as the glycan-originating monosaccharides more likely to be used as substrates for growth of *S*. *pneumoniae* D39 in the respiratory tract.

The predicted routes for Gal, GlcNAc and Man dissimilation in *S*. *pneumoniae* D39 (Fig 1) were validated at the biochemical and genetic level in this study.

### Mannose

The phosphorylated intermediate Man6P accumulated to high levels in exponential cells and the activity of Man6P isomerase (ManA) was induced by Man. A *manA* mutant was unable to grow on Man, even though mannose 6-phosphate isomerase activity was detected to a certain extent in the loss-of-function mutant. Homologues of genes coding for ManA or other known Man6P isomerases were not found in the genome of D39, suggesting that the residual isomerase activity is non-specific, and insufficient to support growth on Man, as shown in Fig 6D. Of interest, a strain expressing *manA* in *trans* under the control of an inducible zinc promoter showed better growth than the wild type D39, suggesting a bottleneck at the level of ManA. This observation is further supported by the high accumulation of Man6P during growth on Man.

### N-acetylglucosamine

Both intermediates for the catabolism of GlcNAc, GlcNAc6P and GlcN6P, were present in exponentially-growing cells. Furthermore, the activity of GlcNAc6P deacetylase was induced by GlcNAc. A *nagA* mutant lost the ability to grow on the amino sugar, and the growth could be restored by in *trans* complementation. In other bacteria GlcNAc can be taken up via PTS or non-PTS transporters [73–78]. A recent report indicated that *S*. *pneumoniae* internalizes this amino sugar exclusively via PTS transporter(s) [8], and this has been confirmed for strain D39 (A. M. Cavaleiro, P. Gaspar, T. Kloosterman, O. P. Kuipers and A. R. Neves, unpublished data).

### Galactose

In a previous study using a different isolate of *S*. *pneumoniae* D39, we reported activity of both the Leloir and the T6P pathways in the utilization of Gal, since intermediates of both pathways (α-Gal1P and α-Glc1P from the Leloir and TBP from the T6P pathway) were observed during growth on Gal [9], and this was confirmed with D39 isolate used in the present study. Specific activities of enzymes for the Leloir (galactokinase, GalK) or T6P (tagatose 1,6-diphosphate aldolase, LacD) pathways were detected during growth on Gal, but not on Glc. This result for GalK and LacD contrasts with the activities of the other enzymes tested (NagA and ManA), which showed activity also when grown on Glc and not only on their dedicated sugars (Table 4). These findings may be explained by the fact that both NagA and ManA are involved in cellular processes other than the catabolism of the monosaccharides, such as providing precursors for biosynthesis. Inactivation of both *galK* and *lacD* in D39 rendered the pneumococcus unable to grow on Gal. Surprisingly, inactivation of *galK* alone abolished growth on Gal, whereas exponential growth of a *lacD* mutant was observed although only after a long lag phase. The behavior of the *lacD* mutant can be partially explained in the context of carbon catabolite repression. The Leloir genes are under strong negative control by the carbon catabolite protein A, CcpA [9], thus alleviation of the repression is required before the pathway is activated. In line with this conclusion, in D39 Glc-grown cells the activity of galactokinase was undetectable. Catabolism via the Leloir pathway is normally associated with uptake of Gal via a non-PTS permease (secondary carriers or ABC transporters). Bidossi *et al*. [8], implicated the ABC transporter SPD_0088-9-90 CUT1 in the uptake of Gal. On the other hand, a *Lactococcus lactis* strain exclusively harboring the Leloir pathway, inactivated for the Gal permease, translocates Gal by a PTS and the resulting Gal6P enters the Leloir pathway upon dephosphorylation by a phosphatase [79]. The same could be active in *S*. *pneumoniae*, however, exclusive transport via the PTS is not in agreement with the observations that *S*. *pneumoniae* G54 and DP1004 devoid of PTS activity (*ptsI* mutants) are capable of growing on Gal [8]. Interestingly, a D39 *ptsI* mutant shows good growth on Gal, but only after a lag phase of about 11 h (A. M. Cavaleiro, P. Gaspar, T. Kloosterman, O. P. Kuipers and A. R. Neves, unpublished data). In light of these results, we propose that induction of an adequate transport system, presumably a non-PTS type, and alleviation of the carbon catabolite repression exerted over the Leloir genes are strict requirements for Gal dissimilation via the Leloir pathway in *S*. *pneumoniae* D39. Our hypothesis is in agreement with the elimination of the lag phase upon subculturing of the *lacD* strain on Gal containing-medium (S6 Fig).

While the residual growth on Gal in the *lacD* mutant can be attributed to activation of the Leloir pathway, we do not know why growth on Gal is totally abolished in the *galK* mutant. A plausible explanation relies on the unintentional elimination of Gal1P uridylyltransferase (GalT) activity that provides essential precursors for the biosynthesis of structural polysaccharides. Indeed, inactivation of *galK* affected the expression of *galT-2*, and reversion of the *galK* mutant to the wild type phenotype on Gal required complementation with both *galK* and *galT-2*. However, inability to grow on Gal could be largely attributed to the *galK* mutation, since expression in *trans* of *galT-2* did not restore growth and a *galT-2* mutant was able to grow on Gal (data not shown). In the latter mutant, expression of *galT-1* was substantially increased, indicating that the product of the duplicated gene accounts for the lost GalT-2 activity, thus restoring the Leloir pathway functionality. Based on our data, we therefore conclude that Gal metabolism in *S*. *pneumoniae* requires an operational Leloir pathway, or at least an active galactokinase.

In other streptococci, efficient dissimilation of Gal has been associated with the presence of a high affinity specific Gal transporter [80,81]. According to Bidossi *et al*. [8], the PTS transporters implicated in the import of Gal in *S*. *pneumoniae* are the mannose-family PTS (ManMNL), a galactitol-family PTS (SPD_0559-0-1) and probably a mannose-family PTS (SPD_0066-7-8-9). In addition, our team showed that *lacFE* genes are induced by Gal, suggesting a potential contribution of the lactose-PTS to the uptake of the sugar [9]. Interestingly, the galactitol-family PTS, which has previously been implicated in Gal uptake [8,53], is a homologue of the specific galactose-PTS identified in *Streptococcus gordonii*, *Streptococcus mutans* and *Streptococcus oligofermentans* [80–82]. However, the affinity of this putative galactose-specific PTS for Gal is seemingly not very high, as noted by markedly decreased growth rates at low Gal concentration and the inability to fully scavenge Gal from the culture medium (Fig 3 and Table 2). The absence of high-affinity PTSs would certainly be a bottleneck for efficient functionality of the T6P pathway, but cannot fully explain the *galK* phenotype. Moreover, the uptake of Gal via a PTS system ensures a typical PTS-mediated signal transduction pathway for CcpA regulation and renders Gal an effective inducer of catabolite repression [9]. Of note, our team has previously reported that CcpA repression of key metabolic genes (Leloir pathway and fermentative pathways) is counterbalanced by a Gal-dependent activation [9]. In view of the results with the *galK* mutant we propose that galactokinase activity is essential for Gal catabolism, and that its product, α-Gal1P, is likely to be the inducer of gene expression. Collectively, our data provide evidence that utilization of Gal in *S*. *pneumoniae* is subject to complex regulation and of a subtle regulatory link between the Leloir and the T6P pathways. Unravelling these regulatory mechanisms certainly deserves future investigations.

Growth in non-preferential sugars is usually associated with mixed acid fermentation profiles [83,84]. This is indeed the case for *S*. *pneumoniae* D39 growing on Gal as sole carbon source (Table 3; [9]). Unexpectedly, growth on the other slowly metabolizable monosaccharide, Man, resulted in only a modest shift towards mixed acid products suggesting a different regulation of the central carbon pathways in the presence of Man. The underlying mechanisms are, however, out of the scope of this work and will be further investigated in the future.

We hypothesised that the ability to efficiently use monosaccharides originating from mucins conferred on *S*. *pneumoniae* a metabolic advantage during colonisation and subsequent invasive states. *S*. *pneumoniae* strains defective in Gal catabolic genes, and in particular the double mutant, D39Δ*lacD*Δ*galK*, presented impaired ability to colonise the murine nasopharynx and had reduced virulence. Inactivation of *nagA* or *manA* had no significant effect in test mice. Importantly, direct administration of the Gal mutants into the bloodstream induced responses of the same magnitude (similar survival times and CFU per ml of blood) as the wild type D39 strain. In the bloodstream the main sugar present is Glc [10,11], and thus the Gal-deficient phenotype was not expected to have any impact. Furthermore, this finding adds to the importance of Gal metabolism in the airways, further supporting the view connecting Gal metabolism in *S*. *pneumoniae* to its virulence in this niche.

At first glance, the inefficient Gal metabolism and the observation that Gal is a less preferred sugar compared to Man and GlcNAc *in vitro*, seems to be in conflict with the role of Gal genes in colonisation and virulence. The lack of correlation between sugar preferences *in vitro* and the effect of mutations in specific sugar catabolic pathways *in vivo* has been reported before for *E*. *coli* [85]. This apparent inconsistency might arise from the multifactorial milieu in host niches. Furthermore, we showed that Gal catabolic genes represent the largest fraction of genes induced by mucin and others had previously established that Gal is widespread and abundant in the airway glycoconjugates (*e*.*g*. mucins). The relevance of Gal acquisition and metabolism had been previously suggested. Indeed, loss of beta-galactosidase activity, resulted in attenuated pneumococcal growth in the nasopharynx [14], and mutants defective in pyruvate formate lyase, an enzyme essential for Gal fermentation, were attenuated in virulence [39]. Furthermore, using Tn-seq in *S*. *pneumoniae* TIGR4 van Opijnen *et al*. [86] identified *lacD* as relevant for pneumococcal fitness in the nasopharynx. In addition, they also showed that both *galK* and *lacD* played a critical role for fitness of TIGR4 during *in vitro* growth on Gal. In *S*. *pneumoniae* D39, *galK* is essential for growth on Gal, while loss of *lacD* causes a long lag phase prior to exponential growth. The different results in the two studies most likely derive from using different serotypes (D39 *vs*. TIGR4) or different experimental conditions for growth of *S*. *pneumoniae*. Nevertheless, both studies highlight the relevance of Gal and its catabolism in the airways.

In light of these results we propose that pneumococcal Gal metabolism is of key importance during colonisation and throughout the transition from carriage to an invasive state. The loss of fitness in the Gal mutants can be due to metabolic impairment and deficient expression of specific virulence traits induced by Gal. We have previously reported that Gal-grown cells produce twice as much capsule as Glc-grown cells [9], and it has been reported that a thicker capsule allows evasion from immune system and from initial mucociliary clearance *in vivo* [87]. On Gal, the carbon balance was lower than on other sugars, but no other end-products/metabolites were detected, thus we propose that Gal is directed to processes other than fermentation such as the polysaccharide synthesis [9]. Whether it is the capsule or other factors such as increased carbon availability that determines the Gal-associated virulence remains to be investigated.

In summary, we followed a multidisciplinary approach to identify the monosaccharides in host glycoproteins that serve as carbon sources for growth of *S*. *pneumoniae* strain D39. Accumulating evidence connects pathogenesis to carbohydrate metabolism, and the findings herein presented further strengthen this view as we specifically show that mutants in Gal catabolic genes showed attenuated ability to colonise and reduced virulence following intranasal infection in mouse models. With widespread antibiotic resistance and re-emergence of non-type vaccine strains, it is urgent that new targets are found for the development of novel therapeutic and preventive drugs. One such opportunity is perhaps offered by the discovery of Gal as an “essential” nutrient for pneumococcal growth and persistence in the host. The Leloir and the tagatose 6-phosphate pathways are present and conserved across pneumococcal serogroups (S8 Table) supporting our suggestion that Gal catabolism could be a potential target for novel therapeutics.

## Supporting Information

S1 Fig Schematic representation of N-acetylglucosamine 6-phosphate synthesis. N-acetylglucosamine 6-phosphate *4* was obtained through a modification of established procedures [46,47]. Benzylation of the anomeric hydroxyl group afforded compound *2* [47], which was selectively phosphorylated at the primary hydroxyl group using dibenzyldiisopropyl phosphoramidite and pyridinium hydrochloride followed by oxidation of the resulting phosphite, with cumene hydroperoxide, to the corresponding phosphate *3*. Hydrogenolysis of *3* with H_2_/Pd/C (10%) in ethanol/water afforded N-acetylglucosamine 6-phosphate *4* quantitatively. Interestingly, when the hydrogenation was performed under anhydrous conditions it was not possible to remove the benzyl protecting group at the anomeric position.
(TIF)

S2 Fig Schematic representation of tagatose 1,6-biphosphate 11 synthesis. Modifications to the synthesis previously published [48] were made in order to optimize the process (details in S1 Text). 1,2:3,4-di-*O*-isopropylidene-α-D-tagatofuranose *6* was obtained in one step (78%) from D-tagatopyranose [Jenkinson et al., 2011], instead of the 4 steps required when starting from D-galacturonic acid. Phosphorylation of *6*, under the same reaction conditions used for the synthesis of *3* (S1 Fig), afforded protected tagatose 6-phosphate *7* in 94% yield. Hydrogenolysis of the benzyl protecting groups of the phosphate afforded phosphate *8* and partially hydrolysed compound *9*, which was probably catalysed by the acidic hydrogen phosphate group. However, this was not a problem, since the next step was the removal of the isopropylidene acetals to afford tagatose 6-phosphate *10*. The enzymatic phosphorylation [88] of the primary alcohol at C-1 of *10* afforded tagatose 1,6-bisphosphate in 66% yield, as the salt of triethylammonium. All attempts to chemically phosphorylate the C-1 primary alcohol failed.
(TIF)

S3 Fig Genomic environment of target genes. (*A*) Schematic representation of the genomic context of sugar pathway mutant strains generated in this study. Mutants were constructed by allelic replacement mutagenesis. (*B*) Organization of genes involved in the catabolism of galactose (A), mannose (B) and N-acetylglucosamine (C). Genes are represented by large arrows. Genes known to be involved in the catabolic reactions (S4 Table) are shown in black, while other genes in the same operons are shown in grey. Promoters (arrows) and terminators (lollipops) were determined by RNA-seq [89]. Gene annotations (as from NCBI): *galR*, galactose operon repressor; g*alK*, galactokinase; *galT-2*, galactose 1-phosphate uridylyltransferase; *lacC*, tagatose 6-phosphate kinase; *lacD*, tagatose 1,6-diphosphate aldolase; *lacT*, transcription antiterminator LacT; SPD_1867, hypothetical protein; *nagA*, N-acetylglucosamine 6-phosphate deacetylase; SPD_1865, zinc-containing alcohol dehydrogenase; SPD_0640, pseudo; *manA*, mannose 6-phosphate isomerase; SPD_0642, sodium-dependent transporter; *agaS*, sugar isomerase; *galM*, aldose 1-epimerase; SDP_1617, cell wall surface anchor family protein; SPD_1616, hypothetical protein; SPD_1615, hypothetical protein; SPD_1614, phosphate transport system regulatory protein PhoU; *galT-1*, galactose 1-phosphate uridylyltransferase; *galE-2*, UDP-glucose 4-epimerase; SPD_1611, hypothetical protein; SPD_1610, hypothetical protein; SPD_1609, ABC transporter substrate-binding protein; SPD_1608, ABC transporter ATP-binding protein; SPD_1607, ABC transporter permease; SPD_1606, MgtC/SapB family protein; *galE-1*, UDP-glucose 4-epimerase; SPD_1431, glycosyl transferase family protein; *fer*, ferredoxin; SPD_1429, hypothetical protein; *cmk*, cytidylate kinase; *pgm*, phosphoglucomutase/phosphomannomutase family protein; *lacA*, galactose 6-phosphate isomerase subunit LacA; *lacB*, galactose 6-phosphate isomerase subunit LacB; *nagB*, glucosamine 6-phosphate isomerase; *rpsU*, 30S ribosomal protein S21. Spe^R^ spectinomycin resistance marker.
(TIF)

S4 Fig Growth profiles of the sugar specific deletion mutants and their complemented derivatives in C+Y medium. (*A*) Growth on galactose (Gal) of D39Δ*galK* complemented with pKB01-*galKgalT-2*. (*B*) Growth on galactose (Gal) of D39Δ*lacD* complemented with pKB01-*lacD*. (*C*) Growth on N-acetylglucosamine (GlcNAc) of D39Δ*nagA* complemented with pKB01*-nagA*. (*D*) Growth on mannose (Man) of D39Δ*manA* complemented with pKB01*-manA*. Growths were made in C+Y (without sucrose and glucose) with or without 0.1 mM ZnCl_2_, at 37ºC. Symbols: (dash) D39 grown in presence of zinc; (grey triangle) D39 loss-of-function mutants grown in presence of zinc; (closed circle) complemented strains grown in presence of zinc; (open circle) complemented strains grown without Zn; (cross) D39 grown without Zn; (open triangle) D39Δ*nagA* grown without Zn.
(TIF)

S5 Fig Analysis of pneumococcal strains in bacteremia model. Mice were infected intravenously with 100 μl PBS containing approximately 5 X10^5^ CFU through dorsal tail vein. (*A*) The animals were monitored over 168 h. Symbols show the times when individual mice became severely lethargic, the point when the animals were culled. The horizontal bars mark the median times to the severely lethargic state. (*B*) Growth of bacteria in the blood. Each point is the mean of data from five mice. Error bars show the standard error of the mean.
(TIF)

S6 Fig Subculturing of *S*. *pneumoniae* D39Δ*lacD* in chemically defined medium (CDM) supplemented with galactose (Gal). (*A*) Pre-culture of *S*. *pneumoniae* D39Δ*lacD* in CDM-Gal (55 mM). 1 ml glycerol stock inoculated in 80 ml CDM-Gal (see Materials and Methods). (*B*) Subculturing of *S*. *pneumoniae* D39Δ*lacD*. Inoculation of fresh CDM-Gal (30 mM), to an initial optical density at 600 nm (OD_600_) of ~ 0.05, with a pre-culture grown in the same sugar (Gal) until late-exponential phase of growth. Growth was performed at 37ºC, under semi-anaerobic conditions, without pH control (initial pH 6.5).
(TIF)

S1 Table Bacterial strains and plasmids used in this study. (DOCX)

S2 Table Pairwise comparisons, across combinations of different sugars and initial substrate concentrations, to test the null hypothesis of equal values for the μ_max_ and OD_max._
(DOCX)

S3 Table Oligonucleotide primers used in this study (from 5’ to 3’). (DOCX)

S4 Table Genes proposed to be involved in the uptake and dedicated catabolism of galactose (Gal), mannose (Man), N-acetylneuraminic acid (NeuNAc), N-acetylglucosamine (GlcNAc), fucose (Fuc), and glucose (Glc) in *S*. *pneumoniae* D39. (DOCX)

S5 Table Expression levels of genes upregulated in *S*. *pneumoniae* D39 cells grown in mucin as compared to glucose-grown cells, according to the established criterion (see Materials and Methods). (DOCX)

S6 Table Expression ratio as determined by qRT-PCR of genes selected from the microarray experiment comparing mRNA levels in mucin-grown to glucose-grown *S*. *pneumoniae* D39 cells. (DOCX)

S7 Table Expression ratio of genes involved in galactose catabolism in exponentially growing *S*. *pneumoniae* D39 cells disrupted in *galK* or *galT-2* genes. (DOCX)

S8 Table Galactose metabolic genes in different *S*. *pneumoniae* strains. (XLSX)

S1 Text Modifications to the previously published synthesis of tagatose 1,6-biphosphate. (DOCX)

S2 Text Genomic potential for the utilization of host monosaccharides. (DOCX)
