# Correction: Host Glycan Sugar-Specific Pathways in *Streptococcus pneumonia*: Galactose as a Key Sugar in Colonisation and Infection

**DOI:** 10.1371/journal.pone.0127483

**Published:** 2015-04-30

**Authors:** The PLOS ONE Staff

The word “*pneumoniae”* is misspelled in the article title. The correct title is: Host Glycan Sugar-Specific Pathways in *Streptococcus pneumoniae*: Galactose as a Key Sugar in Colonisation and Infection. The correct citation is: Paixão L, Oliveira J, Veríssimo A, Vinga S, Lourenço EC, Ventura MR, et al. (2015) Host Glycan Sugar-Specific Pathways in *Streptococcus pneumoniae*: Galactose as a Key Sugar in Colonisation and Infection. PLoS ONE 10(3): e0121042. doi:10.1371/journal.pone.0121042. The publisher apologizes for the error.
