# Supplementary figures and images for: Host Glycan Sugar-Specific Pathways in Streptococcus pneumonia: Galactose as a Key Sugar in Colonisation and Infection
Source: PLoS One. 2015 Mar 31;10(3):e0121042. doi: 10.1371/journal.pone.0121042 (PMC4380338; doi:10.1371/journal.pone.0121042)

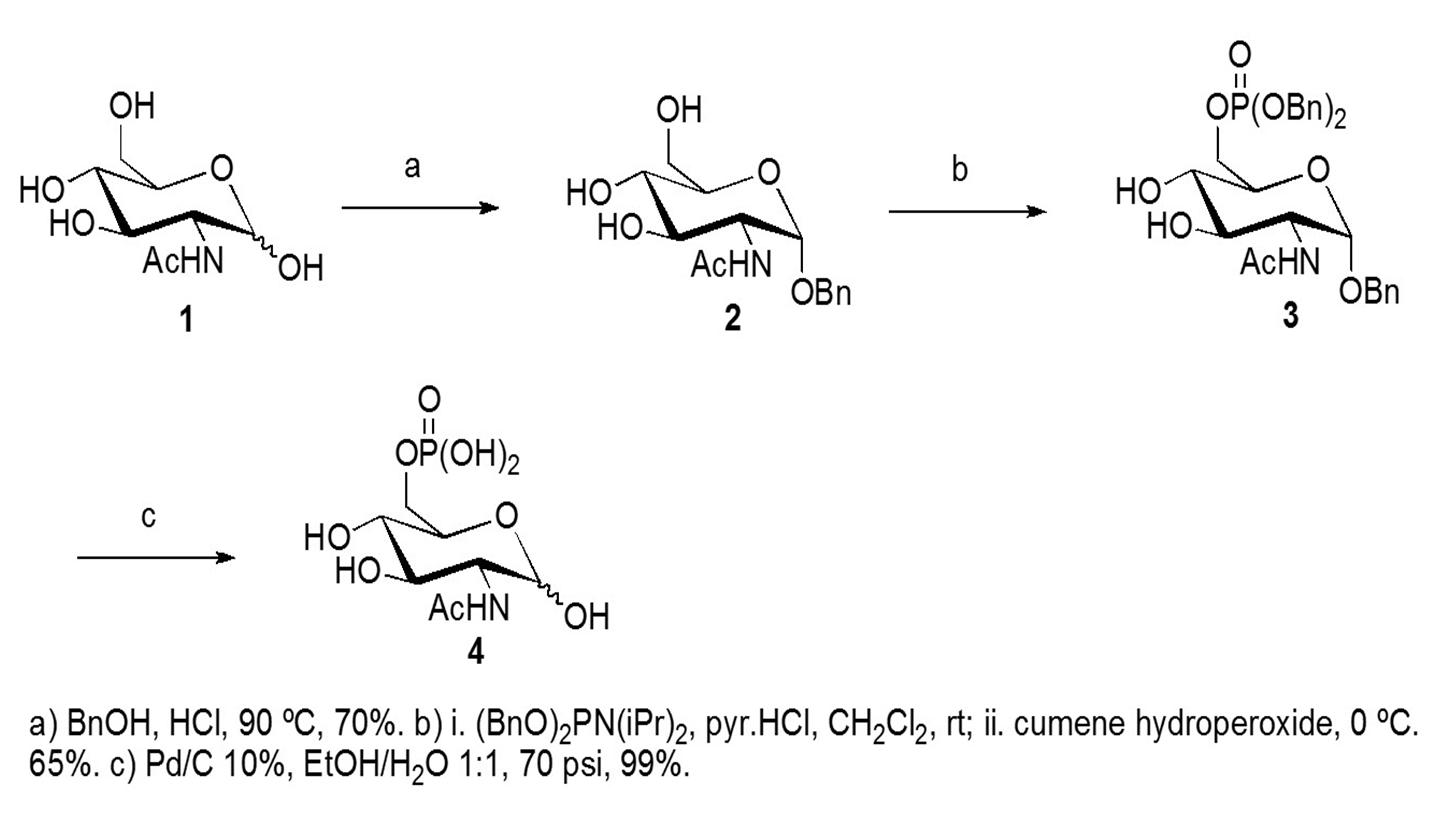

Supplement: S1 Fig — N-acetylglucosamine 6-phosphate 4 was obtained through a modification of established procedures [46,47]. Benzylation of the anomeric hydroxyl group afforded compound 2 [47], which was selectively phosphorylated at the primary hydroxyl group using dibenzyldiisopropyl phosphoramidite and pyridinium hydrochloride followed by oxidation of the resulting phosphite, with cumene hydroperoxide, to the corresponding phosphate 3. Hydrogenolysis of 3 with H2/Pd/C (10%) in ethanol/water afforded N-acetylglucosamine 6-phosphate 4 quantitatively. Interestingly, when the hydrogenation was performed under anhydrous conditions it was not possible to remove the benzyl protecting group at the anomeric position. (TIF) [file pone.0121042.s001.tif]

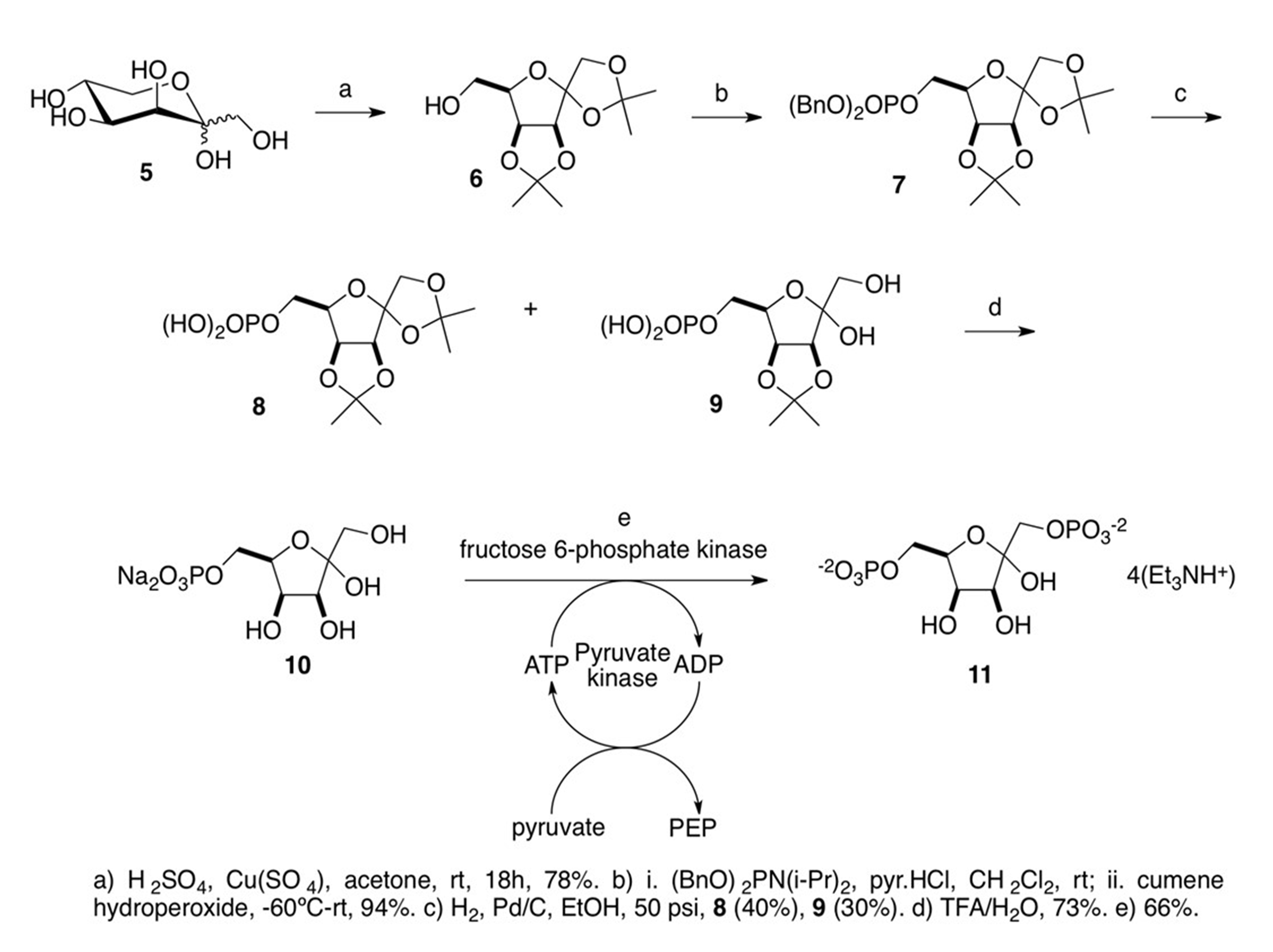

Supplement: S2 Fig — Modifications to the synthesis previously published [48] were made in order to optimize the process (details in S1 Text). 1,2:3,4-di-O-isopropylidene-α-D-tagatofuranose 6 was obtained in one step (78%) from D-tagatopyranose [Jenkinson et al., 2011], instead of the 4 steps required when starting from D-galacturonic acid. Phosphorylation of 6, under the same reaction conditions used for the synthesis of 3 (S1 Fig), afforded protected tagatose 6-phosphate 7 in 94% yield. Hydrogenolysis of the benzyl protecting groups of the phosphate afforded phosphate 8 and partially hydrolysed compound 9, which was probably catalysed by the acidic hydrogen phosphate group. However, this was not a problem, since the next step was the removal of the isopropylidene acetals to afford tagatose 6-phosphate 10. The enzymatic phosphorylation [88] of the primary alcohol at C-1 of 10 afforded tagatose 1,6-bisphosphate in 66% yield, as the salt of triethylammonium. All attempts to chemically phosphorylate the C-1 primary alcohol failed. (TIF) [file pone.0121042.s002.tif]

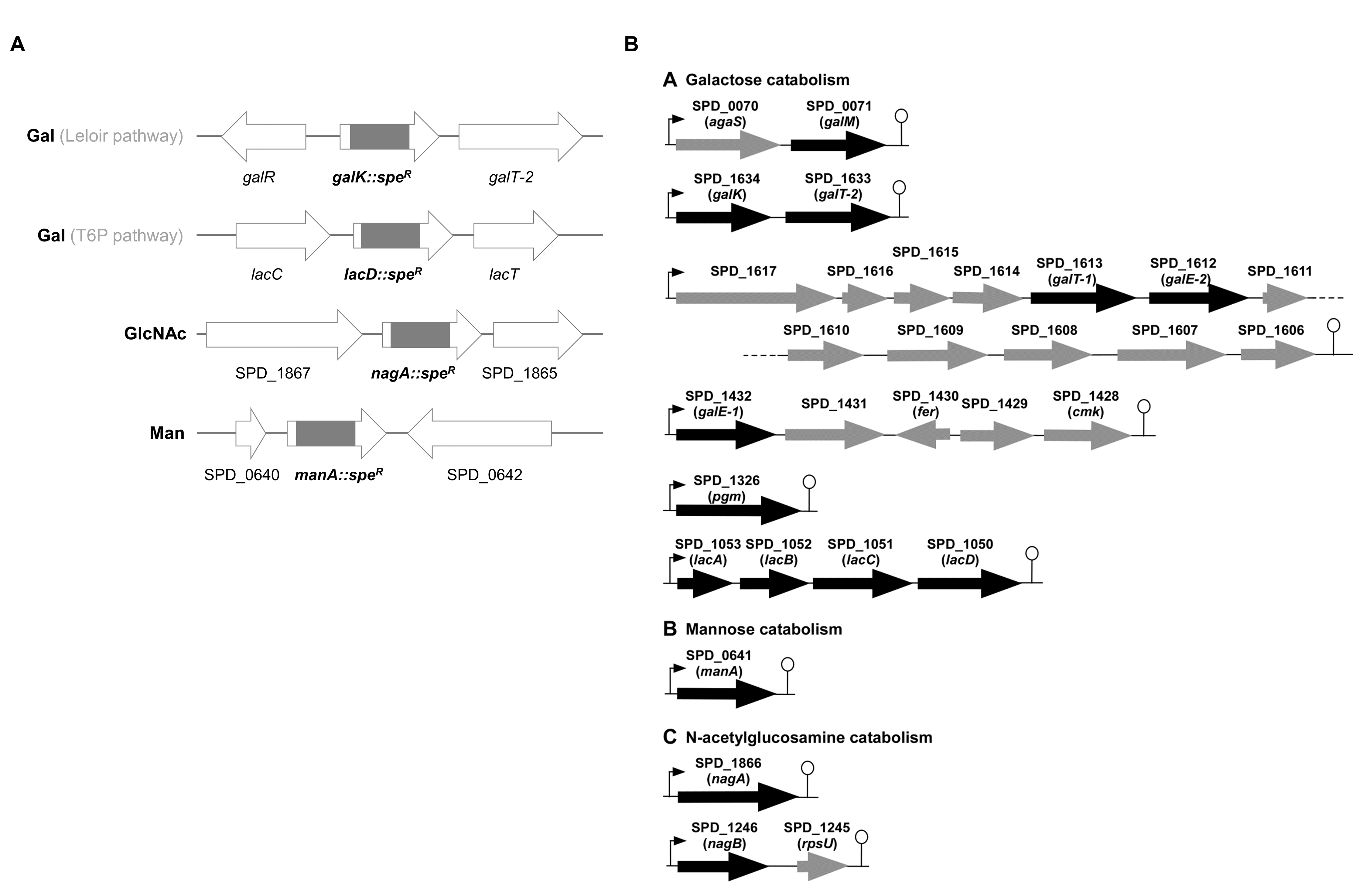

Supplement: S3 Fig — (A) Schematic representation of the genomic context of sugar pathway mutant strains generated in this study. Mutants were constructed by allelic replacement mutagenesis. (B) Organization of genes involved in the catabolism of galactose (A), mannose (B) and N-acetylglucosamine (C). Genes are represented by large arrows. Genes known to be involved in the catabolic reactions (S4 Table) are shown in black, while other genes in the same operons are shown in grey. Promoters (arrows) and terminators (lollipops) were determined by RNA-seq [89]. Gene annotations (as from NCBI): galR, galactose operon repressor; galK, galactokinase; galT-2, galactose 1-phosphate uridylyltransferase; lacC, tagatose 6-phosphate kinase; lacD, tagatose 1,6-diphosphate aldolase; lacT, transcription antiterminator LacT; SPD_1867, hypothetical protein; nagA, N-acetylglucosamine 6-phosphate deacetylase; SPD_1865, zinc-containing alcohol dehydrogenase; SPD_0640, pseudo; manA, mannose 6-phosphate isomerase; SPD_0642, sodium-dependent transporter; agaS, sugar isomerase; galM, aldose 1-epimerase; SDP_1617, cell wall surface anchor family protein; SPD_1616, hypothetical protein; SPD_1615, hypothetical protein; SPD_1614, phosphate transport system regulatory protein PhoU; galT-1, galactose 1-phosphate uridylyltransferase; galE-2, UDP-glucose 4-epimerase; SPD_1611, hypothetical protein; SPD_1610, hypothetical protein; SPD_1609, ABC transporter substrate-binding protein; SPD_1608, ABC transporter ATP-binding protein; SPD_1607, ABC transporter permease; SPD_1606, MgtC/SapB family protein; galE-1, UDP-glucose 4-epimerase; SPD_1431, glycosyl transferase family protein; fer, ferredoxin; SPD_1429, hypothetical protein; cmk, cytidylate kinase; pgm, phosphoglucomutase/phosphomannomutase family protein; lacA, galactose 6-phosphate isomerase subunit LacA; lacB, galactose 6-phosphate isomerase subunit LacB; nagB, glucosamine 6-phosphate isomerase; rpsU, 30S ribosomal protein S21. SpeR spectinomycin resistance marker. [file pone.0121042.s003.tif]

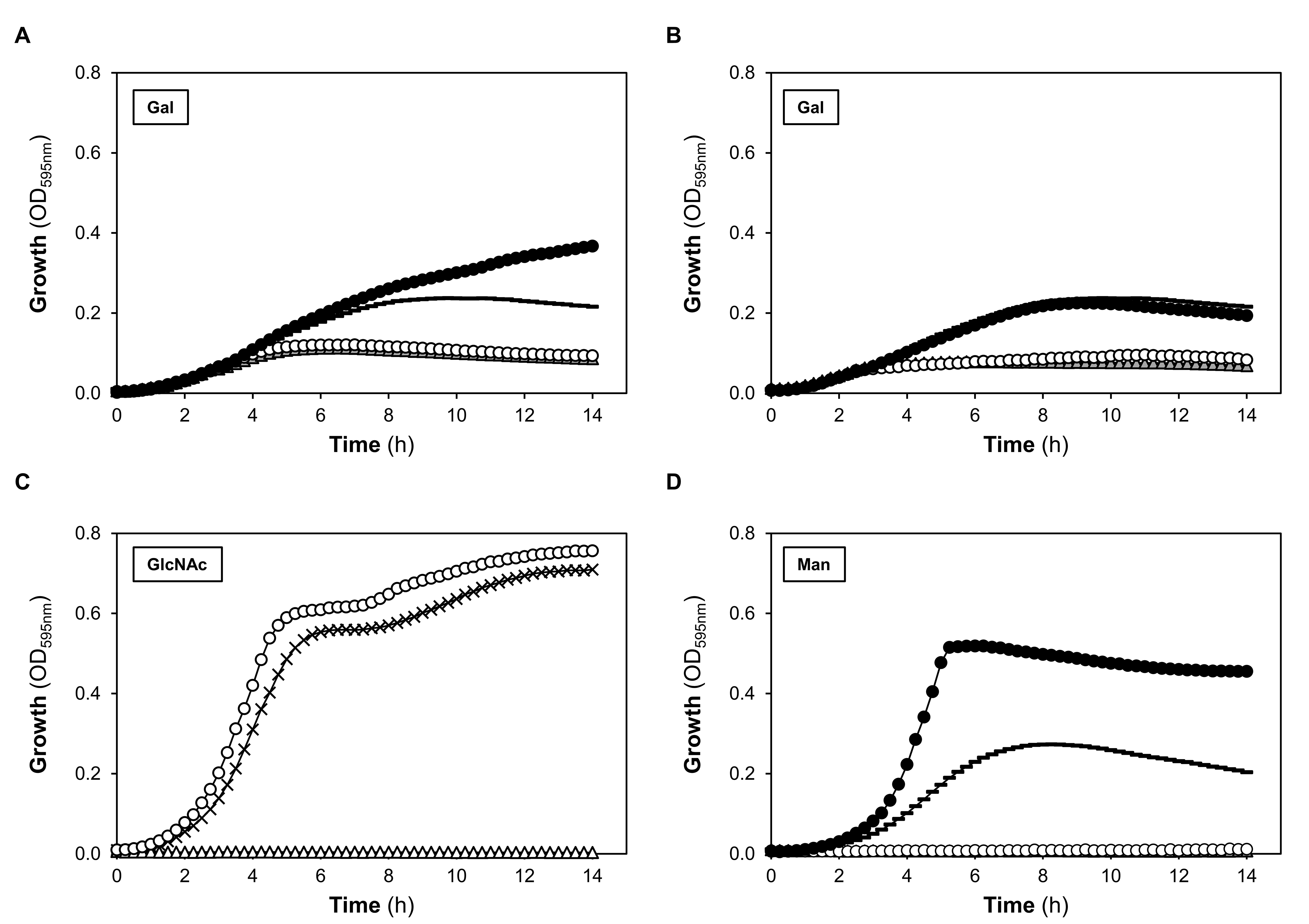

Supplement: S4 Fig — (A) Growth on galactose (Gal) of D39ΔgalK complemented with pKB01-galKgalT-2. (B) Growth on galactose (Gal) of D39ΔlacD complemented with pKB01-lacD. (C) Growth on N-acetylglucosamine (GlcNAc) of D39ΔnagA complemented with pKB01-nagA. (D) Growth on mannose (Man) of D39ΔmanA complemented with pKB01-manA. Growths were made in C+Y (without sucrose and glucose) with or without 0.1 mM ZnCl2, at 37ºC. Symbols: (dash) D39 grown in presence of zinc; (grey triangle) D39 loss-of-function mutants grown in presence of zinc; (closed circle) complemented strains grown in presence of zinc; (open circle) complemented strains grown without Zn; (cross) D39 grown without Zn; (open triangle) D39ΔnagA grown without Zn. (TIF) [file pone.0121042.s004.tif]

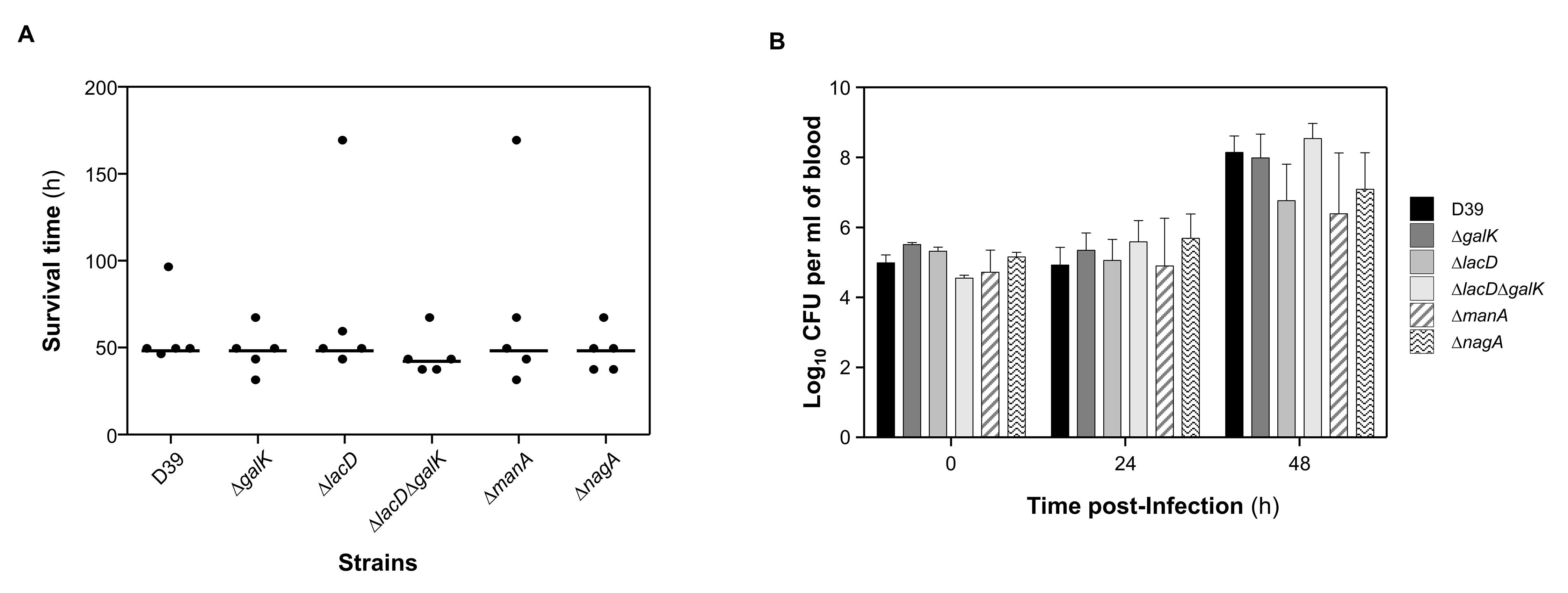

Supplement: S5 Fig — Mice were infected intravenously with 100 μl PBS containing approximately 5 X105 CFU through dorsal tail vein. (A) The animals were monitored over 168 h. Symbols show the times when individual mice became severely lethargic, the point when the animals were culled. The horizontal bars mark the median times to the severely lethargic state. (B) Growth of bacteria in the blood. Each point is the mean of data from five mice. Error bars show the standard error of the mean. (TIF) [file pone.0121042.s005.tif]

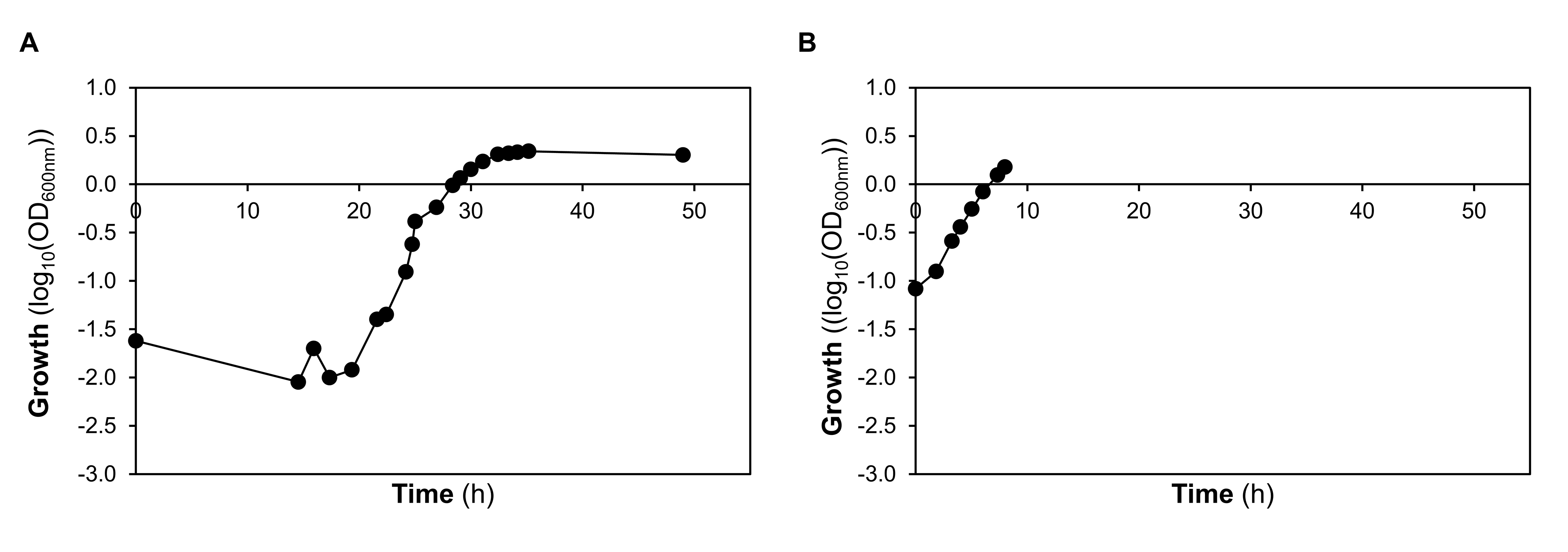

Supplement: S6 Fig — (A) Pre-culture of S. pneumoniae D39ΔlacD in CDM-Gal (55 mM). 1 ml glycerol stock inoculated in 80 ml CDM-Gal (see Materials and Methods). (B) Subculturing of S. pneumoniae D39ΔlacD. Inoculation of fresh CDM-Gal (30 mM), to an initial optical density at 600 nm (OD600) of ~ 0.05, with a pre-culture grown in the same sugar (Gal) until late-exponential phase of growth. Growth was performed at 37ºC, under semi-anaerobic conditions, without pH control (initial pH 6.5). (TIF) [file pone.0121042.s006.tif]
