# Supplementary material for: Host Glycan Sugar-Specific Pathways in Streptococcus pneumonia: Galactose as a Key Sugar in Colonisation and Infection
Source: PLoS One. 2015 Mar 31;10(3):e0121042. doi: 10.1371/journal.pone.0121042 (PMC4380338; doi:10.1371/journal.pone.0121042)
Supplement: S1 Table — (DOCX) [file pone.0121042.s007.docx]

**S1 Table. Bacterial strains and plasmids used in this study.**

| **Strain** | **Relevant genotype** | **Reference/Source** |
| --- | --- | --- |
| ***S. pneumoniae*** |  |  |
| D39 | serotype 2 strain, *cps2* | (a) |
| D39∆*galK* | D39 ∆*galK::spe*, Spe^R^ | This work |
| D39∆*lacD* | D39 ∆*lacD::spe,* Spe^R^ | This work |
| D39∆*manA* | D39 ∆*manA::spe,* Spe^R^ | This work |
| D39∆*nagA* | D39 ∆*nagA::spe,* Spe^R^ | This work |
| D39∆*lacDΔgalK* | D39 ∆*lacD::spe* ∆*galK::tmp,* Spe^R^*,* Tmp^R^ | This work |
| D39∆*galT-2* | D39 ∆*galT-2::spe*, Spe^R^ | This work |
| D39∆*galK* + pKB01-*galK* | D39 ∆*galK::spc, bgaA::*P*_zn_-galK-sfgfp(iGEM)*, Spe^R^,Tet^R^ | This work |
| D39∆*lacD* + pKB01-*lacD* | D39 ∆*lacD::spc, bgaA::*P*_zn_-lacD-sfgfp(iGEM),* Spe^R^,Tet^R^ | This work |
| D39∆*manA* + pKB01-*manA* | D39 ∆*manA::spc, bgaA::*P*_zn_-manA-sfgfp(iGEM)*,Spe^R^,Tet^R^ | This work |
| D39∆*nagA* + pKB01-*nagA* | D39 ∆*nagA::spc, bgaA::*P*_ownnagA_-nagA-sfgfp(iGEM)*, Spe^R^,Tet^R^ | This work |
| D39∆*galK* + pKB01-*galT-2* | D39 ∆*galK::spc, bgaA::*P*_Zn_-galT-2-sfgfp(iGEM)*, Spe^R^,Tet^R^ | This work |
| D39∆*galK* + pKB01-*galKgalT-2* | D39 ∆*galK::spc, bgaA::*P*_Zn_-galKgalT-2-sfgfp(iGEM)*, Spe^R^,Tet^R^ | This work |
| ***E. coli*** |  |  |
| DH5α | *F– Φ80*lac*ZΔM15 Δ(*lac*ZYA-*arg*F) U169*rec*A1*end*A1*hsd*R17 (rK–, mK+)*pho*A*sup*E44 λ–*thi*-1*gyr*A96*rel*A1* | Invitrogen (b) |
| DH5α + pKB01 | Amp^R^ | (b) |
| **Plasmids** |  |  |
| pORI138 | Ori^+^ repA^-^, deletion derivative of pWV01; Spe^R^ | [1] |
| pKB01 | *bgaA,* P*_zn_-sfgfp(iGEM)+*, Amp^R^, Tet^R^ | [2] |
| pKB01-*galK* | *bgaA,* P*_zn_-galK-sfgfp(iGEM)*, Amp^R^, Tet^R^ | This work |
| pKB01-*lacD* | *bgaA,* P*_zn_-lacD-sfgfp(iGEM)*, Amp^R^, Tet^R^ | This work |
| pKB01-*manA* | *bgaA,* P*_zn_-manA-sfgfp(iGEM)*, Amp^R^, Tet^R^ | This work |
| pKB01-*nagA* | *bgaA,* P*_ownnagA_-nagA-sfgfp(iGEM)*, Amp^R^, Tet^R^ | This work |
| pKB01-*galT-2* | *bgaA,* P*_zn_-galT-2-sfgfp(iGEM)*, Amp^R^, Tet^R^ | This work |
| pKB01-*galKgalT-2* | *bgaA,* P*_zn_-galKgalT-2-sfgfp(iGEM)*, Amp^R^, Tet^R^ | This work |

Spe^R^, spectinomycin resistance marker, Tmp^R^, trimethoprim resistance marker, Amp^R^, ampicillin resistance marker, Tet^R^, tetracycline resistance marker.

**(a)** Laboratory stock obtained from the Department of Infection, Immunity & Inflammation, University of Leicester.

**(b)** Laboratory stock obtained from the Molecular Genetics Department, Groningen Biomolecular Sciences and Biotechnology Institute (GBB), Centre for Synthetic Biology, University of Groningen.

1. Leenhouts K, Buist G, Bolhuis A, ten Berge A, Kiel J, Mierau I, et al. A general system for generating unlabelled gene replacements in bacterial chromosomes. Mol Gen Genet MGG. 1996;253: 217–224.

2. Overkamp W, Beilharz K, Detert Oude Weme R, Solopova A, Karsens H, Kovacs AT, et al. Benchmarking various green fluorescent protein variants in *Bacillus subtilis*, *Streptococcus pneumoniae*, and *Lactococcus lactis* for live cell imaging. Appl Env Microbiol. 2013;79: 6481–6490. doi:10.1128/AEM.02033-13
