# Supplementary material for: Host Glycan Sugar-Specific Pathways in Streptococcus pneumonia: Galactose as a Key Sugar in Colonisation and Infection
Source: PLoS One. 2015 Mar 31;10(3):e0121042. doi: 10.1371/journal.pone.0121042 (PMC4380338; doi:10.1371/journal.pone.0121042)
Supplement: S2 Table — (DOCX) [file pone.0121042.s008.docx]

**S2 Table**. **Pairwise comparisons, across combinations of different sugars and initial substrate concentrations, to test the null hypothesis of equal values for the µ_max_ and OD_max._**

|  |  | **Lower sugar concentration** | | | | **Higher sugar concentration** | | | |
| --- | --- | --- | --- | --- | --- | --- | --- | --- | --- |
| **Sugar A** | **Sugar B** | **n_A_** | **n_B_** | **µ_max_** | **OD_max_** | **n_A_** | **n_B_** | **µ_max_** | **OD_max_** |
| Glc | Gal | 12 | 9 | < 10⁻⁷ | NS | 10 | 10 | < 10⁻⁷ | NS |
| Glc | GlcNAc | 12 | 8 | < 10⁻⁷ | 0.049621 | 10 | 8 | < 10⁻⁷ | 0.000041 |
| Glc | Man | 12 | 14 | < 10⁻⁷ | NS | 10 | 9 | < 10⁻⁷ | 0.000002 |
| Gal | GlcNAc | 9 | 8 | 0.000057 | NS | 10 | 8 | 0.009460 | 0.000329 |
| Gal | Man | 9 | 14 | < 10⁻⁷ | NS | 10 | 9 | NS | 0.000017 |
| GlcNAc | Man | 14 | 8 | 0.008151 | NS | 8 | 9 | 0.001814 | NS |

For each comparison, the *p-values* of the Student’s t-test are reported. Statistically significant values (*p-value*<0.05) are shown. *P-values*>0.05 were considered non-significant (NS). The number of replicates for each condition is depicted (n).
