# Supplementary material for: Host Glycan Sugar-Specific Pathways in Streptococcus pneumonia: Galactose as a Key Sugar in Colonisation and Infection
Source: PLoS One. 2015 Mar 31;10(3):e0121042. doi: 10.1371/journal.pone.0121042 (PMC4380338; doi:10.1371/journal.pone.0121042)
Supplement: S3 Table — (DOCX) [file pone.0121042.s009.docx]

**S3 Table. Oligonucleotide primers used in this study (from 5’ to 3’).**

| **Primer** | **Sequence (from 5’ to 3’)** | **Description** |
| --- | --- | --- |
| Allelic replacement mutagenesis | |  |
| GalK_KO1_Fw | CTAGACGCTTGTCCTGAATG |  |
| GalK_KO2_Rv_Spe | ***TCCTCCTCACTATTTTGATTAG***GCGAAGAGTTTCAGCAGTAAGATG | ***Overlap with* spe** |
| GalK_KO3_Fw_Spe | ***CGTTTTAGCGTTTATTTCGTTTAGT***GTTGCAGGTGGCACTCGCGTC | ***Overlap with* spe** |
| GalK_KO4_Rv | CGGCAGGTCGGCATTAGATCC |  |
| LacD_KO1_Fw | CTCAATTCAGGGAGAAACTCG |  |
| LacD_KO2_Rv_Spe | ***TCCTCCTCACTATTTTGATTAG***GATGATACCATTTTCATCAGAAAG | ***Overlap with* spe** |
| LacD_KO3_Fw_Spe | ***CGTTTTAGCGTTTATTTCGTTTAGT***GCGACTTCATGGAAAGAACGTGTG | ***Overlap with* spe** |
| LacD_KO4_Rv | CAATCGACTCCACAAGTTCCAC |  |
| ManA_KO1_Fw | GACTTTCCTGATAGAGTTGTTC |  |
| ManA_KO2_Rv_Spe | ***TCCTCCTCACTATTTTGATTAG***GGTACCACCCCAGATTTTTTCTTGC | ***Overlap with* spe** |
| ManA_KO3_Fw_Spe | ***CGTTTTAGCGTTTATTTCGTTTAGT***TCTTGGACTCTAGAAGGGC | ***Overlap with* spe** |
| ManA_KO4_Rv | GGTTCTTTACTTCCTCAAACCAG |  |
| NagA_KO1_Fw | CTAAGACGGTGGTCATTGCGACTG |  |
| NagA_KO2_Rv_Spe | ***TCCTCCTCACTATTTTGATTAG***GTGTGGGTAGAAAAACTGATCCGC | ***Overlap with* spe** |
| NagA_KO3_Fw_Spe | ***CGTTTTAGCGTTTATTTCGTTTAGT***GATGGCGTAAAACGTTATCAAGCA | ***Overlap with* spe** |
| NagA_KO4_Rv | CACGTAGATATTCAGCCTGCATACC |  |
| Spe_Fp | CTAATCAAAATAGTGAGGAGG |  |
| Spe_Rp | ACTAAACGAAATAAACGC |  |
| GalK_KO2_Rv_Tmp | ***TCCAAGCTCACAAAAATCC***GCGAAGAGTTTCAGCAGTAAGATG | ***Overlap with* tmp** |
| GalK_KO3_Fw_Tmp | ***CCGTCTATGCGCGTCGTAAC***GTTGCAGGTGGCACTCGCGTC | ***Overlap with* tmp** |
| Tmp_ FW | GGATTTTTGTGAGCTTGGA |  |
| Tmp_RV | GTTACGACGCGCATAGACGG |  |
| GalT-2_KO1_Fw | CTTCAGCATCCTTGGAGATCTTG |  |
| GalT-2_KO2_Rv_Spe | ***TCCTCCTCACTATTTTGATTAG***GACATGTGTTACAAATTTATCTACTAAGGTCAC | ***Overlap with* spe** |
| GalT-2_KO3_FW_Spe | ***CGTTTTAGCGTTTATTTCGTTTAGT***GGAATTTTACTAGACTAG | ***Overlap with* spe** |
| GalT-2_KO4_Rv | CCCACAATTAGGGCAATAAGAC |  |
| Confirmation of genotype by PCR | |  |
| GalK_S_Fw | CCGATAGCTAAACTATCGCTGGC |  |
| GalK_S_Rv | GAAGTCAAGCGTAGGACAGACATTGG |  |
| ManA_S_Fw | GGCGCTTCAACAGTTGATAG |  |
| ManA_S_Rv | GCCATTGCTCTAGGAGCTCAAGCG |  |
| LacD_S_Fw | CGGCTGGTGGTAAGGGACTCAATG |  |
| LacD_S_Rv | CGTATGTTGCGTAATCACATCATAG |  |
| NagA_S_Fw | GTGGGAGCGTTTGAGACAGAC |  |
| NagA_S_Rv | CGCACCGAATGATAGGGCAG |  |
| GalT-2_S_Fw | GGGGTTCTTCATTTCTTGCAAGAAGC |  |
| GalT-2_S_Rv | TGCATAAGCACCATCATTGATTG |  |
| Complementation of loss-of-function mutants | | REnz |
| GalK_Fw_EcoRI | AATTGAATTCCAAAGGAGAAATCATATGACACAACATCTTACTGC | EcoRI |
| GalK_Rv_BamHI | GATCGGATCCCTAGTCAAGGACGCGAGTGCC | BamHI |
| GalK_Fw_EcoRI_B | AATTGAATTCTAAGGAGGCAAATATGACACAACATCTTACTGC | EcoRI |
| GalT-2_Rv_XbaI | ACGTCTAGATGTGCAAGGAGAAAGCTCCT | XbaI |
| LacD_Fw_EcoRI | AATTGAATTCTAAAAGAGGTATAAAATGGCTTTAACAGAAC | EcoRI |
| LacD_Rv_BamHI | GATCGGATCCTTACACACGTTCTTTCC | BamHI |
| NagA_Fw_NotI | aattgcggccgcgagttggagaaatccagc | NotI |
| NagA_Rv_BamHI | GATCGGATCCTTATGCTTGATAACGTTTTACGC | BamHI |
| ManA_Fw_EcoRI | AATTGAATTCAGACAGGAGATTAAGATGTCAGAACCATTATTTTTAC | EcoRI |
| ManA_Rv_BamHI | GATCGGATCCTTATGGATGACTAACAATTAATTC | BamHI |
| GalT-2_Fw_EcoRI | AATTGAATTCAGGAGGCTCTATAGTGACCTTAGTAGATAAATTTG | EcoRI |
| GalT-2_Rv_NotI | GGCCGCGGCCGCCTAGTCTAGTAAAATTCCGACC | NotI |
| Confirmation of integration in the *bgaA* locus of the pneumococcal chromosome | |  |
| Integration 1 | CTTGATGAAACCTACATTTG |  |
| Integration 2 | GCTTCCATTAAGGATAGTTC |  |
| Integration 5 | GCTATCGCTGAGCGCCGG |  |
| Integration 6 | AGCTAGAGTTCCGCAATTGG |  |
| P up gatC Fw | ATGGATGCAATCTTTGACCTAATCGG |  |
| P Tet Rv | CACATCGAAGTGCCGCCAAATCC |  |
| Primers used for qRT-PCR | |  |
| GALT1RTF | CTCGTAAAGTGGACGGGAGA |  |
| GALT1RTR | GCAAGTCCCATCACTTCGAT |  |
| GALT2RTF | TCACACCAATAGCGCGTAAG |  |
| GALT2RTR | AGCCCATGACCTCAATCAAG |  |
| SPD1050F | gccagactgcttggatgttt |  |
| SPD1050R | tcagccacacactcagaacc |  |
| SPD1634F | tctcggtgctcgtatgacag |  |
| SPD1634R | cacctgcaacttcagcgata |  |
| SP0645RTF | GCTTAGAAGCGGATAGTCAAG |  |
| SP0645RTR | GTGAGGAATCGCTACATTTGG |  |
| SP0647RTF | TTGCCACTTGCAGGTATCATC |  |
| SP0647RTR | TTCTGGGAAGGCACCTACAC |  |
| SP1197RTF | CTGGTTCAAAACCAAGGTCTG |  |
| SP1197RTR | GCGGTGCAGGTGTTAACTC |  |
| SP1682RTF | TGATGGGCTGACCAACTG |  |
| SP1682RTR | GTGTTGCCGTGACAGTTG |  |
| SP2184RTF | ACCTGTTGTCCCACCTAGTG |  |
| SP2184RTR | TACTGGACCAGCCATCAAGG |  |
| SP703RTF | TAGCGCCTATAGTGGGTCAG |  |
| SP703RTR | CTGGATTCCAAGAACCTGAAG |  |
| SP1507RTF | ATCGGTTCGAACTCTGGATG |  |
| SP1507RTR | ATAACGCCACCGTTTACTGC |  |
| SP1615RTF | AAAATGTTCGCGTCCGTTAC |  |
| SP1615RTR | GGGTGAGCCGTAACCAATTA |  |
| SP2126RTF | TCATGCCACTTGAAAATCCA |  |
| SP2126RTR | GCCTGAATCGCATCTTCTTC |  |
| SPDRT0709F | AAGGAGACTCAGCTGGTGGA |  |
| SPDRT0709R | CCCATGGCTGTGAAAAGACT |  |

**Abbreviation**s: Spe, spectinomycin; Tmp, trimethoprim. REnz, restriction enzyme. Restriction enzyme sites are underlined.
