# Supplementary material for: Host Glycan Sugar-Specific Pathways in Streptococcus pneumonia: Galactose as a Key Sugar in Colonisation and Infection
Source: PLoS One. 2015 Mar 31;10(3):e0121042. doi: 10.1371/journal.pone.0121042 (PMC4380338; doi:10.1371/journal.pone.0121042)
Supplement: S4 Table — (DOCX) [file pone.0121042.s010.docx]

**S4 Table. Genes proposed to be involved in the uptake and dedicated catabolism of galactose (Gal), mannose (Man), N-acetylneuraminic acid (NeuNAc), N-acetylglucosamine (GlcNAc), fucose (Fuc), and glucose (Glc) in *S. pneumoniae* D39.**

| **Locus_Tag** | **Gene** | **Description** | **Identification Method or Ref.** |
| --- | --- | --- | --- |
| **Galactose** | | | |
| **Gal transport** | | | |
| SPD_0263 | *manM* | PTS system mannose-specific transporter subunit IIC | [1] |
| SPD_0067^a^ | - | PTS system transporter subunit IIC | [1–3] |
| SPD_0561 | *-* | PTS system transporter subunit IIC | [1,4] |
| SPD_0090 | - | ABC transporter substrate-binding protein | [1] |
| **Gal catabolism** | | | |
| SPD_0071 | *galM* | Aldose 1-epimerase | Genome annotation at NCBI |
| SPD_1634 | *galK* | Galactokinase | Genome annotation at NCBI |
| SPD_1613 | *galT-1* | Galactose 1-phosphate uridylyltransferase | Genome annotation at NCBI |
| SPD_1633 | *galT-2* | Galactose 1-phosphate uridylyltransferase | Genome annotation at NCBI |
| SPD_1432 | *galE-1* | UDP-glucose 4-epimerase | Genome annotation at NCBI |
| SPD_1612 | *galE-2* | UDP-glucose 4-epimerase | Genome annotation at NCBI |
| SPD_1326 | *pgm* | Phosphoglucomutase/phosphomannomutase family protein | Genome annotation at NCBI |
| SPD_1053 | *lacA* | Galactose 6-phosphate isomerase subunit LacA | Genome annotation at NCBI |
| SPD_1052 | *lacB* | Galactose 6-phosphate isomerase subunit LacB | Genome annotation at NCBI |
| SPD_1051 | *lacC* | Tagatose 6-phosphate kinase | Genome annotation at NCBI |
| SPD_1050 | *lacD* | Tagatose 1,6-diphosphate aldolase | Genome annotation at NCBI |
| **Mannose** | | | |
| **Man** **transport** | | | |
| SPD_0263 | *manM* | PTS system, mannose-specific transporter subunit IIC | [1] |
| SPD_0428 | *lacE-1* | PTS system, lactose-specific transporter subunit IIBC | [1] |
| SPD_0090 | *-* | ABC transporter, substrate-binding protein | [1] |
| SPD_0067 | *-* | PTS system transporter subunit IIC | Database annotation^b^ |
| SPD_0296 | *-* | PTS system transporter subunit IIC | Database annotation^b^ |
| SPD_1990 | *-* | PTS system transporter subunit IIC | Database annotation^b^ |
| **Man catabolism** | | | |
| SPD_0641 | *manA* | Mannose 6-phosphate isomerase | Genome annotation at NCBI |
| **N-acetylneuraminac acid and N-acetylglucosamine** | | | |
| **NeuNAc transport** | | | |
| SPD_1495 | - | Sugar ABC transporter, sugar-binding protein | [1,5–7] |
| SPD_1502 | - | ABC transporter, substrate-binding protein | [1,6,7] |
| SPD_1170^c^ | - | Oligopeptide ABC transporter, oligopeptide-binding protein | [1,7] |
| **GlcNAc transport** | | | |
| SPD_0263 | *manM* | PTS system, mannose specific transporter subunit IIC | [1] |
| SPD_0661 | *exp5* | PTS system transporter subunit IIABC | Database annotation^b^ |
| SPD_1496 | - | PTS system transporter subunit IIBC | Database annotation^b^ |
| SPD_1532 | - | PTS system IIABC components | Database annotation^b^ |
| SPD_1664 | - | PTS system, trehalose-specific IIABC components | Database annotation^b^ |
| **NeuNAc & GlcNAc catabolism** | | | |
| SPD_1489 | - | N-acetylneuraminate lyase | Genome annotation at NCBI BlastP search^d^ |
| SPD_1163 | - | N-acetylneuraminate lyase | Genome annotation at NCBI BlastP search^d^ |
| SPD_1488 | - | ROK family protein | BlastP search^d^ |
| SPD_1497 | *nanE-1* | N-acetylmannosamine 6-phosphate 2-epimerase | Genome annotation at NCBI |
| SPD_1172 | *nanE-2* | N-acetylmannosamine 6-phosphate 2-epimerase | Genome annotation at NCBI |
| SPD_1171^e^ | *-* | Hypothetical protein | BlastP search^d^ |
| SPD_1866 | *nagA* | N-acetylglucosamine 6-phosphate deacetylase | Genome annotation at NCBI |
| SPD_1246 | *nagB* | Glucosamine 6-phosphate isomerase | Genome annotation at NCBI |
| **Fucose** | | | |
| **Fuc transport** | | | |
| SPD_1990^f^ | - | PTS system, transporter subunit IIC | [1,8] |
| **Fuc catabolism** | | | |
| SPD_1995 | *fucK* | L-fuculose kinase | [8] |
| SPD_1994 | *fucA* | L-fuculose phosphate aldolase | [8] |
| SPD_1986 | *fucI* | L-fucose isomerase | [8] |
| SPD_1993 | *fucU* | RbsD/FucU transport protein family protein | Genome annotation at NCBI |
| **Glucose** | | | |
| **Glc transport** | | | |
| SPD_0263^g^ | *manM* | PTS system, mannose-specific transporter subunit IIC | [1] |
| **Glycolysis** | | | |
| SPD_0580 | *gki* | Glucokinase | Genome annotation at NCBI |
| SPD_1897 | *pgi* | Glucose 6-phosphate isomerase | Genome annotation at NCBI |
| SPD_0789 | *pfkA* | 6-phosphofructokinase | Genome annotation at NCBI |
| SPD_0526 | *fba* | Fructose-bisphosphate aldolase | Genome annotation at NCBI |
| SPD_1404 | *tpiA* | Triosephosphate isomerase | Genome annotation at NCBI |
| SPD_1823 | *gap* | Glyceraldehyde 3-phosphate dehydrogenase | Genome annotation at NCBI |
| SPD_0445 | *pgk* | Phosphoglycerate kinase | Genome annotation at NCBI |
| SPD_1468 | *gpmA* | Phosphoglyceromutase | Genome annotation at NCBI |
| SPD_1012 | *eno* | Phosphopyruvate hydratase | Genome annotation at NCBI |
| SPD_0790 | *pyk* | Pyruvate kinase | Genome annotation at NCBI |

^a^Theoretical Gal transporter. Inferred from genomic context.

^b^According to http://www.membranetransport.org/. For the sake of simplicity, only the genes encoding the EIIC component of the PTS system and the substrate binding protein in case of ABC transporters are shown.

^c^Allelic variation of SP1328 of *S. pneumoniae* TIGR4 [1].

^d^For BlastP searches functionally characterized *E. coli* proteins were used as query.

^e^Homology (22% identity; 39% positives) with YjhT from *E. coli* K-12 MG1655. A recently characterized sialic acid mutarotase [9].

^f^Putative transporter. Fucose transport by this PTS is not yet fully disclosed [1,8,10].

^g^Due to the high multiplicity of glucose transporters of *S. pneumoniae,* only the main glucose uptake system identified in D39 is shown [1].

1. Bidossi A, Mulas L, Decorosi F, Colomba L, Ricci S, Pozzi G, et al. A functional genomics approach to establish the complement of carbohydrate transporters in *Streptococcus pneumoniae*. Miyaji EN, editor. PLoS ONE. 2012;7: e33320. doi:10.1371/journal.pone.0033320

2. Terra VS, Homer KA, Rao SG, Andrew PW, Yesilkaya H. Characterization of novel β-galactosidase activity that contributes to glycoprotein degradation and virulence in *Streptococcus pneumoniae*. Infect Immun. 2010;78: 348–357. doi:10.1128/IAI.00721-09

3. Jeong JK, Kwon O, Lee YM, Oh D-B, Lee JM, Kim S, et al. Characterization of the *Streptococcus pneumoniae* BgaC protein as a novel surface β-galactosidase with specific hydrolysis activity for the Galβ1-3GlcNAc moiety of oligosaccharides. J Bacteriol. 2009;191: 3011–3023. doi:10.1128/JB.01601-08

4. Kaufman GE, Yother J. CcpA-dependent and -independent control of beta-galactosidase expression in *Streptococcus pneumoniae* occurs via regulation of an upstream phosphotransferase system-encoding operon. J Bacteriol. 2007;189: 5183–5192. doi:10.1128/JB.00449-07

5. Marion C, Burnaugh AM, Woodiga SA, King SJ. Sialic acid transport contributes to pneumococcal colonization. Infect Immun. 2011;79: 1262–1269. doi:10.1128/IAI.00832-10

6. King SJ, Hippe KR, Gould JM, Bae D, Peterson S, Cline RT, et al. Phase variable desialylation of host proteins that bind to *Streptococcus pneumoniae* in vivo and protect the airway: Pneumococcal desialylation of host proteins. Mol Microbiol. 2004;54: 159–171. doi:10.1111/j.1365-2958.2004.04252.x

7. Almagro-Moreno S, Boyd EF. Insights into the evolution of sialic acid catabolism among bacteria. BMC Evol Biol. 2009;9: 118. doi:10.1186/1471-2148-9-118

8. Higgins MA, Suits MD, Marsters C, Boraston AB. Structural and functional analysis of fucose-processing enzymes from *Streptococcus pneumoniae*. J Mol Biol. 2014;426: 1469–1482. doi:10.1016/j.jmb.2013.12.006

9. Severi E, Muller A, Potts JR, Leech A, Williamson D, Wilson KS, et al. Sialic acid mutarotation is catalyzed by the *Escherichia coli* beta-propeller protein YjhT. J Biol Chem. 2008;283: 4841–4849. doi:10.1074/jbc.M707822200

10. Higgins MA, Whitworth GE, El Warry N, Randriantsoa M, Samain E, Burke RD, et al. Differential recognition and hydrolysis of host carbohydrate antigens by *Streptococcus pneumoniae* family 98 glycoside hydrolases. J Biol Chem. 2009;284: 26161–26173. doi:10.1074/jbc.M109.024067
