# Supplementary material for: Host Glycan Sugar-Specific Pathways in Streptococcus pneumonia: Galactose as a Key Sugar in Colonisation and Infection
Source: PLoS One. 2015 Mar 31;10(3):e0121042. doi: 10.1371/journal.pone.0121042 (PMC4380338; doi:10.1371/journal.pone.0121042)
Supplement: S5 Table — (DOCX) [file pone.0121042.s011.docx]

**S5 Table. Expression levels of genes upregulated in *S. pneumoniae* D39 cells grown in mucin as compared to glucose-grown cells, according to the established criterion (see Materials and Methods).**

| **Gene** | **Fold** | **P-value** | **Description** |
| --- | --- | --- | --- |
| SPD_0562 | 71.8 | 0.0062 | beta-galactosidase |
| SPD_1057 | 61.3 | 0.0085 | PTS system transporter subunit IIB |
| SPD_0561 | 45.5 | 0.0478 | PTS system transporter subunit IIC |
| SPD_0068 | 25.4 | 0.0206 | PTS system transporter subunit IID |
| SPD_1053 | 24.5 | 0.0201 | galactose-6-phosphate isomerase subunit LacA |
| SPD_0069 | 23.9 | 0.0185 | PTS system transporter subunit IIA |
| SPD_1052 | 21.7 | 0.0307 | galactose-6-phosphate isomerase subunit LacB |
| SPD_2013 | 20.8 | 0.0074 | glycerol kinase |
| SPD_0071 | 19.4 | 0.0281 | aldose 1-epimerase |
| SPD_1050 | 17.6 | 0.0411 | tagatose 1,6-diphosphate aldolase |
| SPD_0559 | 15.1 | 0.0041 | PTS system transporter subunit IIA |
| SPD_0560 | 12.1 | 0.0127 | PTS system transporter subunit IIB |
| SPD_2011 | 10.3 | 0.0031 | glycerol uptake facilitator protein |
| SPD_1494 | 10.3 | 0.0197 | sugar ABC transporter permease |
| SPD_0070 | 9.8 | 0.0393 | sugar isomerase |
| SPD_0610 | 8.9 | 0.0096 | hypothetical protein |
| SPD_0063 | 8.7 | 0.0335 | beta-N-acetylhexosaminidase |
| SPD_1590 | 8.6 | 0.0463 | general stress protein 24 |
| SPD_1663 | 8.0 | 0.0191 | alpha,alpha-phosphotrehalase |
| SPD_0066 | 7.8 | 0.0255 | PTS system transporter subunit IIB |
| SPD_0287 | 7.7 | 0.0017 | hyaluronate lyase |
| SPD_1495 | 6.7 | 0.0252 | sugar ABC transporter sugar-binding protein |
| SPD_0293 | 6.5 | 0.0292 | PTS system transporter subunit IIA |
| SPD_1834 | 5.8 | 0.0216 | bifunctional acetaldehyde-CoA/alcohol dehydrogenase |
| SPD_1977 | 5.6 | 0.0114 | carbamate kinase |
| SPD_0613 | 5.5 | 0.0091 | hypothetical protein |
| SPD_0292 | 5.3 | 0.0093 | gluconate 5-dehydrogenase |
| SPD_0259 | 5.3 | 0.0398 | hypothetical protein |
| SPD_1976 | 5.2 | 0.0018 | ornithine carbamoyltransferase |
| SPD_0337 | 5.2 | 0.0425 | Holliday junction-specific endonuclease |
| SPD_1979 | 5.1 | 0.0009 | hypothetical protein |
| SPD_1652 | 4.9 | 0.038 | iron-compound ABC transporter iron-compound-binding protein |
| SPD_1978 | 4.9 | 0.0063 | hypothetical protein |
| SPD_1409 | 4.8 | 0.0271 | sugar ABC transporter ATP-binding protein |
| SPD_1934 | 4.8 | 0.0048 | maltose/maltodextrin ABC transporter maltose/maltodextrin-binding protein |
| SPD_1975 | 4.7 | 0.0083 | pseudo |
| SPD_0619 | 4.5 | 0.0175 | hypothetical protein |
| SPD_0297 | 4.4 | 0.0419 | PTS system transporter subunit IID |
| SPD_1496 | 4.4 | 0.0193 | PTS system transporter subunit IIBC |
| SPD_1006 | 4.4 | 0.0092 | glucose-1-phosphate adenylyltransferase |
| SPD_0308 | 4.4 | 0.0101 | ATP-dependent Clp protease, ATP-binding subunit |
| SPD_0336 | 4.0 | 0.0238 | penicillin-binding protein 1A |
| SPD_0621 | 4.0 | 0.0104 | lactate oxidase |
| SPD_1935 | 3.9 | 0.0058 | maltodextrin ABC transporter permease |
| SPD_1651 | 3.8 | 0.0115 | iron-compound ABC transporter ATP-binding protein |
| SPD_0420 | 3.7 | 0.021 | formate acetyltransferase |
| SPD_0093 | 3.6 | 0.0308 | hypothetical protein |
| SPD_0095 | 3.6 | 0.0036 | hypothetical protein |
| SPD_0094 | 3.6 | 0.001 | hypothetical protein |
| SPD_1007 | 3.5 | 0.0081 | glucose-1-phosphate adenylyltransferase, GlgD subunit |
| SPD_2012 | 3.4 | 0.0029 | alpha-glycerophosphate oxidase |
| SPD_1408 | 3.3 | 0.0317 | hypothetical protein |
| SPD_1566 | 3.2 | 0.0015 | hypothetical protein |
| SPD_1567 | 3.1 | 0.0153 | thioredoxin |
| SPD_1005 | 2.9 | 0.0272 | glycogen branching protein |
| SPD_1936 | 2.9 | 0.0286 | maltodextrin ABC transporter permease |
| SPD_0250 | 2.9 | 0.038 | pullulanase, extracellular |
| SPD_0086 | 2.8 | 0.0004 | hypothetical protein |
| SPD_0692 | 2.7 | 0.0207 | hypothetical protein |
| SPD_1709 | 2.6 | 0.0298 | chaperonin GroEL |
| SPD_0691 | 2.6 | 0.019 | hypothetical protein |
| SPD_1989 | 2.6 | 0.0438 | PTS system transporter subunit IID |
| SPD_0628 | 2.6 | 0.0281 | transcriptional activator TenA, TENA/THI-4 family protein |
| SPD_0616 | 2.5 | 0.0251 | amino acid ABC transporter ATP-binding protein |
| SPD_0427 | 2.5 | 0.0268 | 6-phospho-beta-galactosidase |
| SPD_1675 | 2.4 | 0.0355 | sugar ABC transporter permease |
| SPD_0925 | 2.4 | 0.0443 | hydrolase |
| SPD_0440 | 2.4 | 0.0139 | hypothetical protein |
| SPD_1789 | 2.4 | 0.0194 | cell wall surface anchor family protein |
| SPD_1937 | 2.3 | 0.0231 | maltodextrose utilization protein MalA |
| SPD_0627 | 2.3 | 0.0275 | hypothetical protein |
| SPD_1554 | 2.2 | 0.0428 | iojap-like protein |
| SPD_0626 | 2.2 | 0.023 | ABC transporter ATP-binding protein |
| SPD_1282 | 2.2 | 0.0192 | hypothetical protein |
| SPD_0424 | 2.2 | 0.0259 | PTS system cellobiose-specific transporter subunit IIC |
| SPD_0814 | 2.2 | 0.004 | agmatine iminohydrolase |
| SPD_1944 | 2.1 | 0.0112 | hypothetical protein |
| SPD_0008 | 2.1 | 0.0103 | hypothetical protein |
| SPD_1557 | 2.1 | 0.0109 | nicotinic acid mononucleotide adenylyltransferase |
| SPD_1558 | 2.1 | 0.0391 | hypothetical protein |
| SPD_0439 | 2.1 | 0.042 | hypothetical protein |
| SPD_1655 | 2.1 | 0.003 | segregation and condensation protein B |
| SPD_0863 | 2.0 | 0.0488 | SsrA-binding protein |
