# Supplementary material for: Host Glycan Sugar-Specific Pathways in Streptococcus pneumonia: Galactose as a Key Sugar in Colonisation and Infection
Source: PLoS One. 2015 Mar 31;10(3):e0121042. doi: 10.1371/journal.pone.0121042 (PMC4380338; doi:10.1371/journal.pone.0121042)
Supplement: S6 Table — (DOCX) [file pone.0121042.s012.docx]

**S6 Table. Expression ratio as determined by qRT-PCR of genes selected from the microarray experiment comparing mRNA levels in mucin-grown to glucose-grown *S. pneumoniae* D39 cells.**

| **Targets** |  | **Relative fold increase** | **SD** |
| --- | --- | --- | --- |
| SPD_0559 | PTS system transporter subunit IIA | 32.9 | 5.0 |
| SPD_0561 | PTS system transporter subunit IIC | 31.4 | 3.1 |
| SPD_0610 | Hypothetical protein | 2.4 | 0.5 |
| SPD_1057 | PTS system transporter subunit IIB | 38.2 | 6.3 |
| SPD_1494 | Sugar ABC transporter permease | 67.0 | 16.9 |
| SPD_2011 | Glycerol uptake facilitator protein | 16.0 | 1.1 |
| SPD_1334 | F0F1 ATP synthase subunit epsilon (EC:3.6.3.14) | 0.3 | 0.1 |
| SPD_1839 | Transketolase (EC:2.2.1.1) | 0.2 | 0.0 |
| SPD_1956 | Dihydroxy-acid dehydratase | 0.2 | 0.1 |

SD, standard deviation.
