# Supplementary material for: Host Glycan Sugar-Specific Pathways in Streptococcus pneumonia: Galactose as a Key Sugar in Colonisation and Infection
Source: PLoS One. 2015 Mar 31;10(3):e0121042. doi: 10.1371/journal.pone.0121042 (PMC4380338; doi:10.1371/journal.pone.0121042)
Supplement: S7 Table — (DOCX) [file pone.0121042.s013.docx]

**S7 Table. Expression ratio of genes involved in galactose catabolism in exponentially growing *S.* *pneumoniae* D39 cells disrupted in *galK* or *galT-2* genes.**

|  | **Carbon source - strain** | | |
| --- | --- | --- | --- |
| **Gene** | **Glc-∆*galK* / Glc-D39** | **Glc-∆*galK* / Gal-D39** | **Gal-∆*galT-2* / Gal-D39** |
| *galT-2* | 0.54 ± 0.24 | 0.51 ± 0.22 | - |
| *galT-1* | ND | ND | 49.41 ± 3.55 |
| *lacD* | 0.81 ± 0.03 | 0.0044 ± 0.0002 | 87.42 ± 5.41 |
| *galK* | - | - | 19.29 ± 0.74 |

Values represent the fold difference ± standard deviation transcript levels in the mutant strain as compared to the wild type. Cells were grown in CDM supplemented with glucose or galactose. In the qRT-PCR experiments, the expression values were normalized relative to the housekeeping gene *gyrB*.

ND, not determined.
