# Supplementary material for: Host Glycan Sugar-Specific Pathways in Streptococcus pneumonia: Galactose as a Key Sugar in Colonisation and Infection
Source: PLoS One. 2015 Mar 31;10(3):e0121042. doi: 10.1371/journal.pone.0121042 (PMC4380338; doi:10.1371/journal.pone.0121042)
Supplement: S1 Text — (DOCX) [file pone.0121042.s015.docx]

**S1 Text.** **Modifications to the previously published synthesis of tagatose 1,6-biphosphate.**

Modifications to the synthesis previously published [1] were made in order to optimize the process.

**General.**

^1^H NMR spectra were obtained at 400MHz in CDCl_3_, with chemical shift values (δ) in ppm downfield from tetramethylsilane, DMSO-d_6_ and in D_2_O, ^13^C NMR spectra were obtained at 100.61 MHz, ^31^P NMR spectra were obtained at 161.97 MHz. Assignments are supported by 2D correlation NMR studies. Medium pressure preparative column chromatography: Silica Gel Merck 60 H. Analytical TLC: Aluminium-backed Silica Gel Merck 60 F254. Reagents and solvents were purified and dried according to Armarego and Chai [2]. All the reactions were carried out under an inert atmosphere (argon).

**6-*O*-diBenzylphospho-(1,2:3,4)-di-*O*-isopropylidene-α-D-tagatofuranose 7.** To a stirred solution of **6** (0.53 g, 2.01 mmol) in THF (10 mL) at r.t. was added dibenzyl *N,N*-diisopropylphosphoramidite (1.33 mL, 4.02 mmol) and tetrazole (0.28 g, 4.02 mmol). After 24 hours and complete conversion of the starting material, the reaction was cooled down to -78ºC and mCPBA (0.69 g, 4.02 mmol) was added. The temperature was allowed to rise to r.t., and after 90 minutes the reaction mixture was evaporated. Purification by flash column chromatography on silica gel (20:80, EtOAc/hexane) afforded the product **6** as a viscous colourless foam (0.98 g, 94%). ^1^H NMR (CDCl_3_): δ 7.37-7.31 (m, 10H, CH_2_Ph), 5.08-5.05 (m, 4H, CH_2_Ph), 4.73 (dd, 1H, J=3.5 Hz, J=5.8 Hz, H-4), 4.58 (d, 1H, J=5.8 Hz, H-3), 4.32-4.12 (m, 3H, H-5, 2xH-6), 4.24 (d, 1H, J=9.7 Hz, H-1), 4.01 (d, 1H, J=9.7 Hz, H-1), 1.42 (s, 3H, OMe), 1.38 (s, 3H, OMe), 1.35 (s, 3H, OMe), 1.26 (s, 3H, OMe). ^13^C NMR (CDCl_3_): δ 135.8, 135.7, 128.7, 128.6, 128.5, 128.0, 127.9 (CH_2_Ph), 112.9 (C-2), 111.9 (C(CH_3_)_2_), 111.8 (C(CH_3_)_2_), 85.0 (C-3), 79.6 (C-4), 77.7 (d, J_C,P_=8.0 Hz, C-5), 69.3 (d, J_C-P_=3.2 Hz, CH_2_Ph), 69.2 (d, J_C-P_ =2.9 Hz, CH_2_Ph), 67.3 (d, J_C-P_=5.7 Hz, C-1), 65.5 (d, J_C-P_=5.4 Hz, C-6) 26.4 (OMe), 26.3 (OMe), 25.9 (OMe), 24.8 (OMe). ^31^P NMR (CDCl_3_): δ -1.15 (s).

**(1,2:3,4)-di-*O*-isopropylidene-α-D-tagatofuranose 6-(dihydrogen phosphate) 8.** Dibenzyl phosphate **7** (0.98 g, 1.88 mmol) was hydrogenated at 50 psi in the presence of Pd/C 10% (0.19 g, 0.18 mmol) in EtOH (12 mL), for 20 hours at r.t. The reaction mixture was then filtered through celite, washed with EtOH and evaporated. Purification by column chromatography on silica gel (60:40, MeOH/CH_2_Cl_2_) afforded phosphate diacetal **8** (0.36 g, 56%) and phosphate monoacetal **9** (α/β 1:8.7, 0.23 g, 41%) as colourless viscous foams. The characterisation data for **8** was identical to the literature [3].

**3,4-*O*-isopropylidene-α/β-D-tagatofuranose 6-(dihydrogen phosphate) 9.** ^1^H NMR (D_2_O) : δ 4.95 (dd, J=3.8 Hz, J=5.9 Hz, H-4 β), 4.91 (dd, J=4.0 Hz, J=6.1 Hz, H-4 α) 4.59 (d, J=5.9 Hz, H-3 β), 4.54 (d, J=5.9 Hz, H-3 α), 4.36-4.32 (m, H-5 β), 4.30-4.27 (m, H-5 α), 4.10-4.04 (m, H-6 β), 3.96-3.90 (m, H-6 β), 3.74 (d, J=11.6 Hz, H-1 β), 3.60 (d, J=11.6 Hz, H-1 β), 1.49 (s, OMe α), 1.41 (s, OMe β), 1.32 (s, OMe α), 1.28 (s, OMe β). ^31^P NMR (D_2_O): δ -0.32 (s).

**D-Tagatose 6-(dihydrogen phosphate).** The product was obtained following the procedure described in the literature [3]. ^1^H NMR (D_2_O): δ 4.29-4.25 (m), 4.23-4.20 (m), 4.11-4.06 (m), 4.04-3.82 (m), 3.51 (dd, J=12.0 Hz, J=16.6 Hz, H-1 α), 3.42 (dd, J=12.0 Hz, J=16.6 Hz, H-1 β). ^13^C NMR (D_2_O): δ 102.7 (C-2), 79.2 (d, J_C,P_=7.9 Hz, C-5), 70.9, 70.7, 64.6 (d, J_C,P_=4.7 Hz, C-6), 62.9 (C-1). ^31^P NMR (D_2_O): δ -0.01 (s).

**D-Tagatose 6-phosphate, disodium salt 10.** The product was obtained following the procedure described in the literature [3]. Yield 73%. ^1^H NMR (D_2_O): δ 4.35-4.32 (m), 4.30-4.28 (m), 4.19-4.11 (m), 4.09-4.00 (m), 3.59 (dd, J=12.0 Hz, J=19.0 Hz, H-1 α), 3.50 (dd, J=12.0 Hz, J=16.4 Hz, H-1 β).

**D-Tagatose 1,6-bisphosphate, tetrakis(triethylammonium) salt 11.** The enzymatic phosphorylation of **10** was accomplished following the procedure described in the literature [3]. The tetrakis(triethylammonium) salt of **11** was prepared instead of the tetrakis(cyclohexylammonium) salt. Anomers α:β 1:4, yield 66%. ^1^H NMR (D_2_O): δ 4.54 (t, 1H, J=5.1 Hz, H-4 α), 4.45-4.42 (m, 1H, H-5 α), 4.38 (dd, 1H, J=4.8, J=4.0 Hz, H-4 β), 2.28 (d, 1H, J=4.9 Hz, H-3 β), 4.25-4.20 (m, 1H, H-5 β), 4.17-4.11 (m, 1H, H-6 β), 4.04-4.00 (m, 1H, H-6 β), 3.88 (dd, 1H, J=10.8, J=5.8 Hz, H-1 β), 3.82 (dd, 1H, J=10.8, J=5.08 Hz, H-1 β), 3.19 (q, 24H, J=7.3 Hz, CH_3_CH_2_N), 1.27 (t, 36H, J=7.3 Hz, CH_3_CH_2_N). ^13^C NMR (D_2_O), Anomer β: δ 101.8 (C-2), 79.04 (d, J=8.1 Hz, C-5), 70.78 and 70.73 (C-3 and C-4), 65.6 (d, J=4.99 Hz, C-1), 64.3 (d, J=5.0 Hz, C-6), 46.6 (CH_3_CH_2_N), 8.2 (CH_3_CH_2_N). ^31^P NMR (D_2_O), Anomer β: δ 0.66 (s), 0.29 (s).

**References**

1. Eyrisch O, Sinerius G, Fessner W-D. Facile enzymic de novo synthesis and NMR spectroscopy characterization of D-tagatose 1,6-biphosphate. Carbohyd Res. 1993;238: 287–306.

2. Armarego WLF, Chai CLL. Purification of laboratory chemicals. 5th ed. Amsterdam ; Boston: Butterworth-Heinemann; 2003.

3. Jenkinson SF, Fleet GWJ, Nash RJ, Koike Y, Adachi I, Yoshihara A, et al. Looking-glass synergistic pharmacological chaperones: DGJ and L-DGJ from the enantiomers of tagatose. Org Lett. 2011;13: 4064–4067. doi:10.1021/ol201552q
