# Supplementary material for: Host Glycan Sugar-Specific Pathways in Streptococcus pneumonia: Galactose as a Key Sugar in Colonisation and Infection
Source: PLoS One. 2015 Mar 31;10(3):e0121042. doi: 10.1371/journal.pone.0121042 (PMC4380338; doi:10.1371/journal.pone.0121042)
Supplement: S2 Text — (DOCX) [file pone.0121042.s016.docx]

#### **S2 Text. Genomic potential for the utilization of host monosaccharides.**

Fucose metabolism

Homologues of genes encoding the *E. coli* Fuc degradation pathway were found in the D39 genome (Fig. 1 and S4 Table) [1–3]. To further metabolize anaerobically the final product of Fuc metabolism, L-lactaldehyde, an L-1,2-propanediol oxidoreductase (FucO) gene is present in some operons [4]. A BlastP search using as query FucO from *E. coli* K-12 MG1655 showed homology (42% identity; 60% positives) to an iron-containing alcohol dehydrogenase (SPD_1985) of D39, but whether this protein is functionally active remains to be elucidated. However, the downstream steps of L-lactaldehyde aerobic degradation seem to be missing in *S. pneumoniae*. A PTS system is present in the Fuc operon of D39, but the implication of this transporter in the uptake of Fuc is not yet proven. It has been proposed to transport Fuc-containing oligosaccharides that would subsequently be processed by intracellular glycoside hydrolases releasing Fuc [2–4].

N-acetylgalactosamine metabolism

Genes involved in the initial steps for the intracellular catabolism of GalNAc [5–7] remain elusive in the pneumococcus (Fig. 1). However, the N-acetylgalactosamine 6-phosphate deacetylase (coded by *agaA*) for conversion of GalNAc6P to GalN6P in *E. coli C* str. ATCC 8739, shares 37% amino acid sequence identity with N-acetylglucosamine 6-phosphate deacetylase (coded by *nagA*) of D39. Recently, it was shown that NagA can substitute the activity of AgaA*,* in *E. coli* [7,8]. Moreover, in this microorganism, it was proposed that the isomeration/deamination of GalN6P to T6P was accomplished by galactosamine 6-phosphate isomerase (coded by *agaS*) and not by *agaI* [7]. The first is annotated in the genome of D39 and encodes a sugar isomerase (45% amino acid sequence identity with the one present in *E. coli* *C* str. ATCC 8739). A putative GalNAc transporter (SPD_0293-5-6-7) is annotated in the D39 genome [3] and shares 33% amino acid sequence identity with the GalNAc transporter subunit EIIC (EcolC_0566) of *E. coli* C str. ATCC 8739. Additionally, the latter shares 32% amino acid sequence identity with SPD_1990, which is annotated as being potentially involved in amino sugar metabolism in D39.

Very recently, the GalNAc pathway was established for *Lactobacillus casei* and is encoded in the *gnb* gene cluster [9]. This route includes a PTS^Gnb^ that transports and phosphorylates the substrate to GalNAc6P and is subsequently processed through GnbF and GnbE, which encode for the activities of GalNAc6P deacetylase and GalN6P deaminase/isomerase, respectively. Moreover, it was found that conversion of GalNAc6P to GalN6P could also be accomplished by NagA, as full growth on GalNAc requires both activities.

The PTS^Gnb^ shares high amino acid sequence homology with the pneumococcal mannose-family PTS SPD_0066-7-8-9. In particular the PTS transport system subunit IIC (LCABL_02930) shares 50% identity and 65% positives with the *S. pneumoniae* D39 protein encoded by SPD_0067. The degradation enzymes GnbF and GnbE share 54% and 48% amino acid sequence identity with NagA (SPD_1866) and AgaS (SPD_0070) of S*. pneumoniae* D39, respectively.

In summary, GalNAc catabolic pathways were established in *E. coli* and *L. casei* and according to our analysis *S. pneumoniae* possesses gene products showing considerable homology to the proteins in these organisms. Therefore, it is tempting to suggest that GalNAc is processed through the combined action of *nagA* and *agaS* in *S. pneumoniae* D39. However, how to reconcile this hypothesis with the inability to grow on GalNAc still remains to be investigated.

N-acetylneuraminic acid metabolism

The NeuNAc utilization has been recently studied [3,10,11].

A duplication event might have occurred for the N-acetylneuraminate lyase gene (SPD_1489 and SPD_1163). N-acetylmannosamine kinase is annotated in the metabolic database MetaCyc as a glucokinase (*gki*). However, a BlastP search led us to suggest that this function is most likely performed by the ROK family protein (SPD_1488) as this locus is part of the *nanAB* operon.

**References**

1. Chan PF, O’Dwyer KM, Palmer LM, Ambrad JD, Ingraham KA, So C, et al. Characterization of a novel fucose-regulated promoter (PfcsK) suitable for gene essentiality and antibacterial mode-of-action studies in *Streptococcus pneumoniae*. J Bacteriol. 2003;185: 2051–2058. doi:10.1128/JB.185.6.2051-2058.2003

2. Higgins MA, Whitworth GE, El Warry N, Randriantsoa M, Samain E, Burke RD, et al. Differential recognition and hydrolysis of host carbohydrate antigens by *Streptococcus pneumoniae* family 98 glycoside hydrolases. J Biol Chem. 2009;284: 26161–26173. doi:10.1074/jbc.M109.024067

3. Bidossi A, Mulas L, Decorosi F, Colomba L, Ricci S, Pozzi G, et al. A functional genomics approach to establish the complement of carbohydrate transporters in *Streptococcus pneumoniae*. Miyaji EN, editor. PLoS ONE. 2012;7: e33320. doi:10.1371/journal.pone.0033320

4. Higgins MA, Suits MD, Marsters C, Boraston AB. Structural and functional analysis of fucose-processing enzymes from *Streptococcus pneumoniae*. J Mol Biol. 2014;426: 1469–1482. doi:10.1016/j.jmb.2013.12.006

5. Reizer J, Ramseier TM, Reizer A, Charbit A, Saier MH Jr. Novel phosphotransferase genes revealed by bacterial genome sequencing: a gene cluster encoding a putative N-acetylgalactosamine metabolic pathway in *Escherichia coli*. Microbiol Read Engl. 1996;142 ( Pt 2): 231–250.

6. Brinkkötter A, Klöss H, Alpert C, Lengeler JW. Pathways for the utilization of N-acetyl-galactosamine and galactosamine in *Escherichia coli*. Mol Microbiol. 2000;37: 125–135.

7. Hu Z, Patel IR, Mukherjee A. Genetic analysis of the roles of *agaA*, *agaI*, and *agaS* genes in the N-acetyl-D-galactosamine and D-galactosamine catabolic pathways in *Escherichia coli* strains O157:H7 and C. BMC Microbiol. 2013;13: 94. doi:10.1186/1471-2180-13-94

8. Leyn SA, Gao F, Yang C, Rodionov DA. N-Acetylgalactosamine utilization pathway and regulon in proteobacteria: genomic reconstruction and experimental characterization in *Shewanella*. J Biol Chem. 2012;287: 28047–28056. doi:10.1074/jbc.M112.382333

9. Bidart GN, Rodríguez-Díaz J, Monedero V, Yebra MJ. A unique gene cluster for the utilization of the mucosal and human milk-associated glycans galacto- *N* -biose and lacto- *N* -biose in *L* *actobacillus casei*: Galacto- and lacto- *N* -biose utilization in *Lactobacillus*. Mol Microbiol. 2014;93: 521–538. doi:10.1111/mmi.12678

10. Gualdi L, Hayre J, Gerlini A, Bidossi A, Colomba L, Trappetti C, et al. Regulation of neuraminidase expression in *Streptococcus pneumoniae*. BMC Microbiol. 2012;12: 200. doi:10.1186/1471-2180-12-200

11. Marion C, Burnaugh AM, Woodiga SA, King SJ. Sialic acid transport contributes to pneumococcal colonization. Infect Immun. 2011;79: 1262–1269. doi:10.1128/IAI.00832-10
